# Building a Physically Interpretable Compressed Chemical Space via Grid-Based Optimization

**DOI:** 10.1021/acs.jcim.6c01542

**Published:** 2026-10-15

**Authors:** Seungmin Yoon, Alex Dickson

**Affiliations:** † Department of Pharmacology and Toxicology, 3078 Michigan State University, East Lansing, Michigan 48824, United States; ‡ Department of Computational Mathematics, Science, and Engineering, Michigan State University, East Lansing, Michigan 48824, United States; § Department of Biochemistry and Molecular Biology, Michigan State University, East Lansing, Michigan 48824, United States

## Abstract

In recent years, deep generative models have been increasingly adopted for molecular optimization and design. In many such models, molecules are encoded into latent spaces that provide compact representations of their features. However, these latent spaces remain difficult to interpret in physical terms, and it is unclear how much structural and molecular information is retained under compression. This limitation becomes especially important when molecular behavior depends on specific three-dimensional arrangements embedded in an environmental context. Here, we introduce COMPRESS (Compression Of Molecular Physical fields into Reduced Spatial Sites), an optimization framework that represents a small molecule using a fixed number of physically parametrized spatial sites. Each site is defined by three spatial coordinates and three interaction parameters, yielding a fixed-size representation that remains directly interpretable in physical terms. To preserve molecular information, COMPRESS representations are optimized to reproduce the density, electrostatic, and van der Waals fields of the corresponding all-atom molecule on discretized three-dimensional grids. We systematically evaluate how molecular information is retained as the number of sites, *K*, is reduced. Our analysis covers benzene, 20 chemically diverse chemical scaffolds, benzene dimers, protein–ligand complexes, and 10,000 molecules chosen from the GEOM-Drugs data set. Across systems, field agreement deteriorates slowly at high resolution and then undergoes a rapid, pronounced transition at lower resolution. We further find that *K* = 25 conformers capture about 97% of the molecules from GEOM-Drugs under a loss threshold of 0.05, which we use as an indicator of fidelity. These results show that COMPRESS can provide a fixed-size, physically interpretable molecular representation, enabling compression to be evaluated directly using molecular interaction fields and resulting physical quantities. We also introduce a decoder that reconstructs all-atom structures directly from a COMPRESS representation, showing that the representation can support molecular generation in addition to field-preservation analysis. Finally, rationally modified COMPRESS representations through changes to site charge and size or the addition or removal of sites are shown to generate realistic chemical compounds upon decoding.

## Graphical abstract

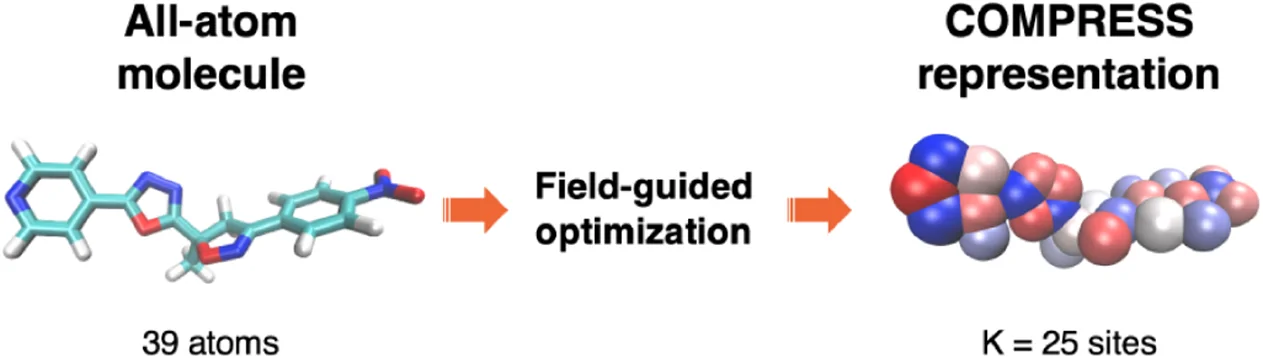

## 1 Introduction

Despite remarkable advances in the life sciences and biotechnology, drug discovery remains a time-consuming and costly process, typically requiring 10 to 15 years and an investment of 1 to 2 billion USD per approved drug.^1,2^ Furthermore, approximately 90% of drug candidates that enter clinical trials ultimately fail to achieve regulatory approval.^1,3^ Computational methods have attracted growing interest because they can prioritize, evaluate, and design candidate molecules more efficiently than traditional experimental workflows.^4^ Most computational approaches have historically concentrated on screening and ranking molecules from existing libraries.^5^ However, despite their increasing size, these libraries cannot come close to capturing the true diversity of drug-like chemical space.^6–8^ Recent research has increasingly focused on the use of generative modeling as a solution to discover new molecules, as these approaches can even identify molecules outside of their large training sets.^9,10^

A central challenge in chemical space exploration is that the discrete nature of molecules makes direct gradient-based optimization difficult.^11^ Deep generative modeling addresses this limitation by encoding molecules into continuous, lower-dimensional latent spaces, where molecular variation can be represented more compactly and explored through generative sampling or optimization.^9,12^ Representative examples include the variational autoencoder (VAE) framework developed by Gómez-Bombarelli et al.^13^ and the latent diffusion model introduced by Zhang et al.^14^ Although these methods have shown promising results in molecular generation, there are several important limitations pertaining to interpretability and controllability.

First, the relationship between learned latent representations and molecular properties or structure is unclear.^15,16^ A shift in latent coordinates reflects coupled changes in both geometry and physicochemical behavior, rather than a single identifiable property.^17^ Relatedly, the extent to which physically meaningful information is retained during compression is undefined, making the physical fidelity of the representation difficult to assess directly. Several studies have explored more interpretable and controllable latent representations. For example, Du et al. identify directions in latent space associated with molecular property improvement,^17^ and Liu et al. decompose the latent space into disentangled components for controllable 3D molecule generation.^18^ Although these studies have improved the interpretability and controllability of latent spaces, fundamentally the generative process still operates as a black box, where only the final output can be evaluated, and no mechanism exists to incorporate physical manipulations during the generative process.

In addition, molecular function in many biologically relevant contexts depends on three-dimensional and environment-responsive organization, particularly within protein-binding pockets.^19^ This challenge is particularly evident in sequence-based models such as MolGPT,^20^ which generate molecules from SMILES strings rather than explicit three-dimensional structures. Although many structure-based generative approaches, such as PocketFlow^21^ and DeepICL,^22^ incorporate spatial environmental information, a recent benchmark of nine pocket-based three-dimensional generative models demonstrated that current methods still produce conformations of poor quality, characterized by high strain energy and substantial steric clashes with protein atoms.^23^ Even if spatial environmental information is included, most generative models treat the protein pocket as a fixed structural context.^24–26^ In nature, proteins are inherently flexible, and different ligands can induce distinct conformations and interaction patterns within a binding pocket.^27,28^ Our previous work on the Flexible Topology framework^29^ addressed this issue by enabling atom-mimicking particles with physically meaningful attributes that dynamically adapt to a protein-binding pocket,^30^ demonstrating that pocket-conditioned spatial and electrostatic templates can be obtained while allowing for an interplay between the binding pocket and the ligand particles. While it was shown that ligand-based virtual screening tools, such as EON (OpenEye Scientific Software),^31^ can be used to identify compounds that match a given template as closely as possible, we hypothesize that a direct generative framework capable of utilizing such pocket-conditioned information will identify better hits at lower computational cost, which constitutes a second motivation for the present work.

In this context, we introduce COMPRESS (Compression Of Molecular Physical fields into Reduced Spatial Sites), an optimization framework that represents each small molecule using a fixed number of physically parametrized spatial sites. Each site is defined by three spatial coordinates, (*x*, *y*, *z*), and three interaction parameters: charge (*q*), Lennard-Jones (LJ) diameter (σ), and well depth (ϵ). A molecular representation is comprised of a fixed set of *K* × 6 parameters, where *K* denotes the number of sites. To preserve physically meaningful information, the reduced representation is optimized to reproduce three molecular fields of the original all-atom molecule: density, electrostatic, and van der Waals (vdW) fields. These fields are represented on discretized three-dimensional grids. The reduced sites are then optimized to match the fields of the target all-atom molecule. COMPRESS therefore provides a fixed-size molecular representation whose elements remain directly interpretable in physical terms, rather than as abstract latent variables.

This approach addresses the limitations of conventional latent representations described above. First, COMPRESS represents molecules as sets of sites with clear physical meaning. Whereas changes in latent space are often difficult to interpret, modifications to a COMPRESS site correspond directly to shifts in position (*x*, *y*, *z*) or physical parameters (*q*, σ, ϵ). This makes the representation both interpretable and controllable, as each parameter fulfills a specific physical role. Second, since the sites are optimized to reproduce spatially distributed molecular fields, the COMPRESS representation directly captures the three-dimensional arrangement of each molecule together with the spatial field patterns that govern its interactions. This allows the representation to retain information relevant to molecular interactions with the surrounding environment. Finally, COMPRESS provides a well-defined measure of compression, *K*, relative to the number of atoms. Systematic variation of *K* enables direct assessment of the amount of field-relevant information retained at different levels of resolution. This approach also enables physical evaluation of compression, for example, by tracking changes in interaction energies and related molecular properties as the resolution decreases.

In this work, we evaluate how well COMPRESS (Figure 1) preserves spatially distributed molecular fields as the number of sites *K* is reduced. The analysis has three parts. First, we use benzene as a minimal reference system and then repeat the same evaluation on 20 chemically diverse scaffolds to assess whether the trends hold across molecules. Second, we perform intermolecular energy analyses of benzene dimers and protein–ligand complexes and track how the interaction energies change with *K*. Third, we conduct a population-level analysis on 10,000 molecules from GEOM-Drugs to quantify how the loss distribution and basic molecular properties shift with *K*.

**Figure 1:**
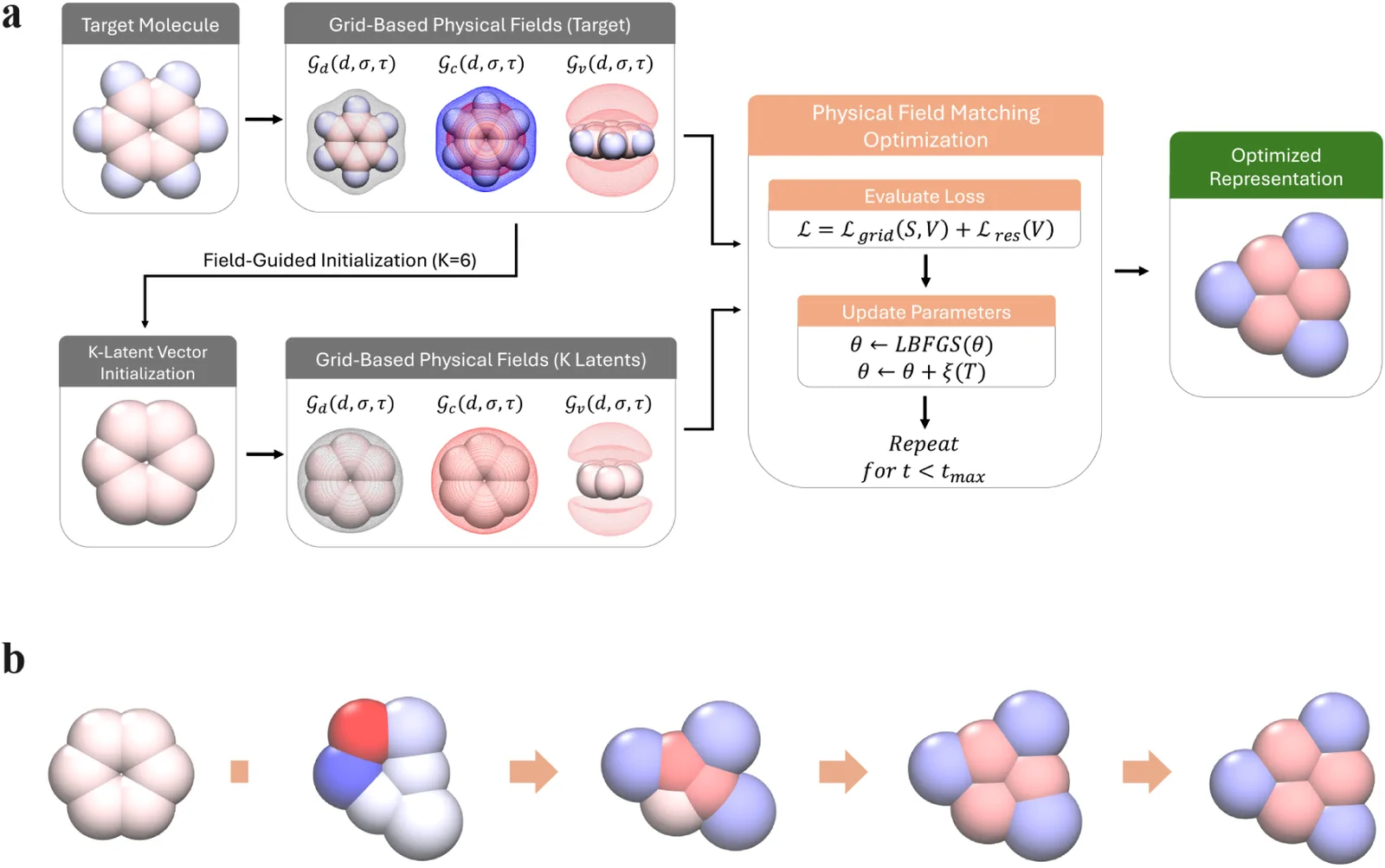
(a) Schematic workflow of the COMPRESS framework, which converts all-atom molecules into a compressed site-based representation. (b) Optimization process of the COMPRESS representation of benzene (K = 6) over 50 steps, from the initial field-guided configuration (step = 0) to the final optimized representation (step = 50). Sites are colored by charge (red: negative, blue: positive) and scaled by LJ radius σ.

As a next step, we introduce a decoder that generates an all-atom structure directly from a COMPRESS representation, to demonstrate how these representations can be used for molecular generation. This decoder is a multimodal flow-matching model, built on the FlowMol3 architecture,^32^ trained to jointly predict atomic positions, partial charges, atom types, and bond orders. Generation of a molecule from a COMPRESS representation yields a valid molecule with the desired shape or charge pattern encoded by the representation. We evaluate the reconstruction fidelity of the decoder across a range of resolution ratios *K*/*M*, and show how it responds to perturbations of the representation in a set of representative examples.

## 2 Methods

### 2.1 Problem Formulation

At the all-atom (AA) level, we describe a molecule with *M* atoms by the set *S* in eq 1,

1 $$S={\{(r_{i},\;q_{i},\;\sigma_{i},\;\epsilon_{i})\}}_{i=1}^{M}$$

where $r_{i}=\left(x_{i},\;y_{i},\;z_{i}\right)\in R^{3}$ is the atomic position, *q*_*i*_ is the partial charge, and (σ_*i*_, ϵ_*i*_) are the Lennard-Jones (LJ) diameter and well depth, respectively.

COMPRESS maps this variable-size description to a fixed-size representation, $S\in R^{M\times 6}\to V\in R^{K\times 6}$ , where *K* < *M* is the number of sites that control the compression level. Each COMPRESS site carries the same parameters ${\{(r_{k},\;q_{k},\;\sigma_{k},\;\epsilon_{k})\}}_{k=1}^{K}$ and is optimized to reproduce the AA molecule’s spatially distributed molecular fields—including density, electrostatic, and van der Waals (vdW) fields—on a three-dimensional grid. The parameters are obtained and optimized by minimizing the discrepancy between the fields induced by *S* and those reconstructed from *V*, as detailed below.

### 2.2 Data Preparation

#### 2.2.1 Parameters for Benzene and Small Scaffold Molecules

We first evaluate COMPRESS by using benzene as well as 20 scaffold molecules identified in the QC-DGE data set.^33^ To obtain chemically specific molecules, each “R” substituent position is functionalized with a carbon atom and associated hydrogen atom(s). Initial three-dimensional coordinates are generated using RDKit^34^ and optimized with the MMFF94 force field.^35^ Physicochemical parameters for field construction, including partial charges and LJ parameters, are obtained with ACPYPE^36^ via AmberTools/Antechamber.^37,38^

#### 2.2.2 Modeling and Equilibration of Protein–Ligand Complexes

To prepare the protein ligand systems for energy calculation, the protein–ligand systems were built using CHARMM-GUI Solution Builder (v3.7).^39,40^ Starting structures were drawn from PDB ID 2RH1 for the β_2_-adrenergic receptor (B2AR) structure with carazolol bound^41^ and 3PTB for trypsin-benzamidine.^42^ Each complex is solvated in a cubic water box under periodic boundary conditions. Water boxes of 71.0 × 71.0 × 71.0 Å^3^ and 144.0 × 144.0 × 144.0 Å^3^ are used for the trypsin and B2AR systems, respectively. To achieve an ionic strength of 0.15 M, 28 K^+^ ions and 36 Cl^–^ ions are added to the trypsin system, and 260 K^+^ and 271 Cl^–^ ions are added to the B2AR system.

Simulations are performed in OpenMM.^43^ After energy minimization, we perform a brief 125 ps of NVT equilibration at 303.15 K using Langevin dynamics. Particle Mesh Ewald is employed for electrostatics, and a force-switching scheme for vdW interactions. To maintain the experimental protein structure during equilibration, harmonic positional restraints are applied to protein backbone and side-chain atoms. The resulting structures were used to calculate interaction energies for protein–ligand systems in the both all-atom and compressed contexts.

#### 2.2.3 Drug-like Molecular Data Set

To evaluate the large-scale behavior of COMPRESS under compression, we use the training split of the GEOM-Drugs data set,^44^ omitting molecules with fewer than 30 atoms or more than 80 atoms. We select the first 10,000 molecules of this set and used five conformers per molecule, giving 50,000 conformations in total. Nonbonded parameters are generated using the same parametrization procedure used for benzene and the 20 scaffold molecules to keep consistency in charge and LJ parameters across all systems.

### 2.3 Grid Representation

Spatially distributed molecular fields derived from *S* are represented by discretizing space with a three-dimensional face-centered cubic (FCC) grid,^45^ which provides more isotropic sampling and reduces directional artifacts in comparison to a simple cubic grid. We define the grid G on a set of FCC grid points $G=\left\{g_{j}\in R^{3}|j=1,\;...,\;N\right\}$ . The grid spacing is set by the nearest-neighbor distance Δ*g* = 0.3 Å for all grids used in this work. The grid boundaries are selected to encompass the spatial extent of *S*, with the grid length along each axis given by *D*_*a*_ = (*r*_max,*a*_ – *r*_min,*a*_) + 2*B*, where *r*_min,*d*_ and *r*_max,*d*_ denote the minimum and maximum atomic coordinates of *S* along axis *a* ∈ {*x*, *y*, *z*}, and *B* = 5 Å is a symmetric buffer added to ensure coverage beyond the outermost atoms. All FCC lattice points are within this bounding volume, resulting in *N* grid points in total.

For each point *g*_*j*_, we compute grid values G(j) by aggregating contributions from the set of atoms *S* or sites *V*. For all field types, the grid values are evaluated at multiple smoothing scales controlled by the parameter τ. Larger τ values yield broader and smoother fields, whereas smaller values emphasize more localized features. The sets of τ used in this work are given in Table 1. We consider three field families on the grid: density, electrostatics, and vdW. The density field at each grid point is defined in eq 2 as the sum of Gaussians centered at the atoms:

2 $$G_{\mathrm{sig}}\left(j;\tau_{\mathrm{sig}}\right)=\underset{i}{\sum }\exp\left(-\frac{d_{ij}^{2}}{2{\left(\tau_{\mathrm{sig}}\sigma_{i}\right)}^{2}}\right)$$

where *d*_*ij*_ = ∥*r*_*i*_ – *g*_*j*_∥_2_ is the Euclidean distance between atom *i* and grid point *j*.

**Table 1:** The Six Fields Used to Optimize COMPRESS Representations, along with Values of the *τ* Hyperparameters Used to Calculate Field Values^a^

Field type	Symbol	Values
Density field	τ_*sig*_	{0.2, 0.5}
Electrostatic field	τ_*ele*_	{0.2, 1.0}
Positive field	τ_*pos*_	{0.2, 1.0}
Negative field	τ_*neg*_	{0.2, 1.0}
Repulsion fields	τ_*rep*_	{0.2, 2.0}
Attration fields	τ_*att*_	{0.2, 2.0}

a Multiple τ values indicate that multiple fields are constructed and used to evaluate the total grid loss.

Second, the electrostatic field induced by partial charges is defined in eq 3:

3 $$G_{\mathrm{ele}}\left(j;\tau_{\mathrm{ele}}\right)=\underset{i}{\sum }\frac{q_{i}}{\sqrt{d_{ij}^{2}+\tau_{\mathrm{ele}}^{2}}}$$

The parameter τ_*ele*_ softens the Coulombic form, keeping the field finite as *d*_*ij*_ → 0. We also separately define positive and negative electrostatic contributions in eqs 4 and 5:

4 $$G_{\mathrm{pos}}\left(j;\tau_{\mathrm{pos}}\right)=\overset{M}{\underset{i}{\sum }}\frac{\max\left(q_{i},\;0\right)}{\sqrt{d_{ij}^{2}+\tau_{\mathrm{pos}}^{2}}}$$

5 $$G_{\mathrm{neg}}\left(j;\tau_{\mathrm{neg}}\right)=\overset{M}{\underset{i}{\sum }}\frac{\min\left(q_{i},\;0\right)}{\sqrt{d_{ij}^{2}+\tau_{\mathrm{neg}}^{2}}}$$

Third, the vdW field is also represented by separate repulsive and attractive terms in eqs 6 and 7:

6 $$G_{\mathrm{rep}}\left(j;\tau_{\mathrm{rep}}\right)=\underset{i}{\sum }4\epsilon_{i}\exp\left(-\frac{d_{ij}^{2}}{2{\left(\tau_{\mathrm{rep}}\sigma_{i}\right)}^{2}}\right)$$

7 $$G_{\mathrm{att}}\left(j;\tau_{\mathrm{att}}\right)=\underset{i}{\sum }4\epsilon_{i}\exp\left(-\frac{{\left(d_{ij}-2^{1/6}\sigma_{i}\right)}^{2}}{2{\left(\tau_{\mathrm{att}}\sigma_{i}\right)}^{2}}\right)$$

The repulsive term is a Gaussian centered at each atom’s position, while the attractive term is a Gaussian centered at a distance of 2^1/6^σ from the atom, corresponding to the location of that atom’s optimal vdW interaction distance.^46^ Both the attractive and repulsive fields are modulated by the LJ energy well-depth for each atom, ϵ_*i*_. The parameters above were determined through iterative optimization considering performance on interaction energies for benzene and the protein–ligand examples.

### 2.4 Optimization

Given *S*, we initialize *V* by selecting atoms that are most influential on the grid fields with the fixed τ = 1.0. For each atom, we compute its contributions to the density, electrostatic, vdW-repulsive, and vdW-attractive fields over all grid points, and sum these values to obtain scores per atom. To normalize the four field scores, each score is divided by the maximum value observed across atoms for each field. The normalized scores are then summed to form a single total score for each atom. Atoms are removed in order of increasing score until *K* atoms remain, defining the $V_{\mathrm{init}}={\{(r_{k},\;q_{k},\;\sigma_{k},\;\epsilon_{k})\}}_{k=1}^{K}$ . *V* is then optimized by following gradients of the total loss $L_{\mathrm{total}}$ , which is defined as in eq 8:

8 $$L_{\mathrm{total}}\left(t\right)=L_{\mathrm{grid}}\left(S,\;V,\;t\right)+L_{\mathrm{restraint}}\left(V,\;t\right)$$

where *t* is the time step of the optimization process and $L_{\mathrm{grid}}$ is the grid loss defined in eqs 9 and 10:

9 $$L_{\mathrm{grid}}\left(S,\;V,\;t\right)=\underset{f\in F}{\sum }\underset{\tau \in T_{f}}{\sum }\lambda_{f}\left(\tau ,\;t\right)L_{\mathrm{norm}}\left(G_{f}^{S},\;G_{f}^{V}\right)$$

10 $$L_{\mathrm{norm}}\left(G_{f}^{S},\;G_{f}^{V}\right)=\frac{{∥G_{f}^{S}-G_{f}^{V}∥}_{2}^{2}}{{∥G_{f}^{S}∥}_{2}^{2}+{∥G_{f}^{V}∥}_{2}^{2}}$$

where $L_{\mathrm{norm}}$ is the normalized loss between two grids $G_{f}^{S}$ and $G_{f}^{V}$ given a field type *f* taken from the set of fields: F={sig,net,pos,neg,rep,att} . The set $T_{f}$ represents the set of τ values used for the field type *f*. The weight λ_*f*_(τ, *t*) depends on both τ and *t* as shown in eq 11:

11 $$\lambda_{f}\left(\tau ,\;t\right)=\alpha_{f}\cdot \min\left(\frac{\left(t+1\right)\tau }{2},\;1\right)$$

with scaling factor α_*f*_, which is 1.5 for vdW grid fields and 1.0 for others. This schedule prioritizes larger τ fields early in optimization and gradually increases the contribution of smaller τ fields as *t* grows. The restraint loss $L_{\mathrm{restraint}}$ enforces *V* to be within physically reasonable parameter ranges. It is described in detail in Appendix A.

The gradients $\nabla_{\theta }L_{\mathrm{total}}$ are evaluated and used to update θ using the L-BFGS optimizer with a strong Wolfe line search.^47^ We set maximum iteration = 20 and history size = 20. After each step, we add Gaussian noise according to eq 12,

12 $$\theta \leftarrow \theta +\sqrt{2\gamma T_{t}}\xi ,,\xi ∼N\left(0,\;I\right)$$

where γ = 10^–7^. The temperature is initialized at *T*_0_ = 1 and is halved every three steps.

All computations used Python 3.10.16 with PyTorch 2.7.1, NumPy 1.26.4, SciPy 1.15.2, RDKit 2024.03.6,^34^ ACPYPE,[Bibr ref36] 2023.10.27, and OpenMM 8.1.2;^43^ visualization used VMD 1.9.4a57.^48^ Runs are performed on NVIDIA GeForce RTX 2080 Ti GPUs with CUDA 12.8. A single COMPRESS optimization (50 steps) completes in approximately 30 s to 1 min per conformer depending on *K*. Optimizing a single conformer across all four resolutions (*K* = 10, 15, 20, and 25) takes under 2 min in total. The full GEOM-Drugs data set (50,000 conformers at four resolutions) required approximately 1,500 GPU-hours.

### 2.5 COMPRESS Decoder

#### 2.5.1 Architecture

To evaluate the potential of COMPRESS representations for molecular design, we introduce a decoder network Ψ_θ_ that maps a COMPRESS representation back into an all-atom structure. Given a COMPRESS representation $V={\{(r_{k},\;q_{k},\;\sigma_{k},\;\epsilon_{k})\}}_{k=1}^{K}$ and a target atom count *M*, the decoder outputs a reconstructed structure $Ŝ={\{({\mathrm{r̂}}_{i},\;{\mathrm{q̂}}_{i},\;{â}_{i})\}}_{i=1}^{M}$ , where *r̂*_*i*_, *q̂*_*i*_, and *â*_*i*_ are the reconstructed atomic position, partial charge, and element identity of atom *i*, together with a symmetric bond-order matrix $\mathrm{B̂}={\{{\mathrm{b̂}}_{ij}\}}_{i,j=1}^{M}\in Z^{M\times M}$ , where *b̂*_*ij*_ is the predicted bond order between atoms *i* and *j*.

Ψ_θ_ is a multimodal flow-matching model,^49^ adapted from FlowMol3,^32^ built on a simpler E­(n)-equivariant graph neural network (EGNN) in place of FlowMol3′s Geometric Vector Perceptron (GVP)-based network. Both the COMPRESS representation *V* and the candidate structure *S* are represented as graphs, *G*_*V*_ and *G*_*S*_. *G*_*V*_ provides conditioning information to *G*_*S*_ and remains static, whereas *G*_*S*_ is iteratively refined through message passing layers in the EGNN. Additional details are given in Appendix B. After the final message passing block, three heads read out the updated atom/edge features: a position/charge head, an atom-type classification head, and a bond-order classification head.

#### 2.5.2 Training

We train Ψ_θ_ with continuous flow matching for atomic positions and partial charges, and discrete flow matching for atom type and bond order. Given a paired sample (*S*_0_, *S*_1_ = *S*), where *S*_0_ is the source atom cloud and *S*_1_ is the target structure, the continuous state at time *t* is linearly interpolated as *S*_*t*_ = (1 – (1 – ν)*t*)*S*_0_ + *t S*_1_, jointly over position and charge, with a small path-noise term ν = 0.01 that keeps the state slightly stochastic. Intermediate values of the discrete atom type and bond order variables are generated according to eqs 13 and 14:^50,51^

13 $$a_{i}^{\left(t\right)}=\{\begin{array}{ll} a_{i}, & \mathrm{with}\;\mathrm{probability}\;t,\\ \mathrm{MASK}, & \mathrm{with}\;\mathrm{probability}\;1-t, \end{array}$$

14 $$b_{\mathrm{ij}}^{\left(t\right)}=\{\begin{array}{ll} b_{\mathrm{ij}}, & \mathrm{with}\;\mathrm{probability}\;t,\\ \mathrm{MASK}, & \mathrm{with}\;\mathrm{probability}\;1-t, \end{array}$$

Training requires a one-to-one atom correspondence between *S*_0_ and *S*_1_ so that *S*_*t*_ interpolates meaningfully between them. Because *S*_0_ is generated from the physically meaningful sites of *V*, it already shares roughly the same shape as *S*, so we couple them with a linear assignment (Hungarian algorithm)^52^ over position and charge alone, without any rotational alignment. The assignment π* is calculated using eq 15:

15 $$\pi^{*}=\arg\underset{\pi \in \Pi_{M}}{\min}\overset{M}{\underset{i=1}{\sum }}C_{i,\pi (i)}$$

where Π_*M*_ is the set of all permutations of *M* atoms and the cost *C*_*ij*_ in eq 16 combines the position and charge distance between candidate atom *i* and target atom *j*:

16 $$C_{ij}=\frac{{∥r_{i}^{\left(0\right)}-r_{j}∥}^{2}}{Z_{r}}+w_{q}\frac{{\left(q_{i}^{\left(0\right)}-q_{j}\right)}^{2}}{Z_{q}}$$

where *Z*_*r*_ and *Z*_*q*_ normalize the position and charge cost terms, as defined in eqs 17 and 18 with charge weight *w*_*q*_ = 0.05.

17 $$Z_{r}=\frac{1}{M^{2}}\overset{M}{\underset{i,j=1}{\sum }}{∥r_{i}^{\left(0\right)}-r_{j}∥}^{2}$$

18 $$Z_{q}=\frac{1}{M^{2}}\overset{M}{\underset{i,j=1}{\sum }}{\left(q_{i}^{\left(0\right)}-q_{j}\right)}^{2}$$

*S* and its bond matrix are reordered according to π* to form the paired sample (*S*_0_, *S*_1_) used to construct *S*_*t*_.

The training objective is the weighted sum in eq 19,

19 $$L=\lambda_{\mathrm{pos}}L_{\mathrm{pos}}+\lambda_{\mathrm{charge}}L_{\mathrm{charge}}+\lambda_{\mathrm{atom}}L_{\mathrm{atom}}+\lambda_{\mathrm{bond}}L_{\mathrm{bond}}$$

with (λ_pos_, λ_charge_, λ_atom_, λ_bond_) = (3.0, 1.0, 0.4, 2.0). The four terms are defined in eq 20:

20 $$\begin{array}{ll} L_{\mathrm{pos}} & =\frac{1}{3M}\overset{M}{\underset{i=1}{\sum }}{∥{\mathrm{r̂}}_{i}-r_{i}∥}^{2},\\ L_{\mathrm{charge}} & =\frac{1}{M}\overset{M}{\underset{i=1}{\sum }}{\left({\mathrm{q̂}}_{i}-q_{i}\right)}^{2},\\ L_{\mathrm{atom}} & =-\frac{1}{\left|A_{\mathrm{mask}}\right|}\underset{i\in A_{\mathrm{mask}}}{\sum }\log\mathrm{p̂}\left(a_{i}|S_{t},\;V\right),\\ L_{\mathrm{bond}} & =-\frac{1}{\left|B_{\mathrm{mask}}\right|}\underset{\left(i,j\right)\in B_{\mathrm{mask}}}{\sum }w_{b_{ij}}\log\mathrm{p̂}(b_{ij}|S_{t},\;V), \end{array}$$

where $A_{\mathrm{mask}}$ and $B_{\mathrm{mask}}$ denote the sets of atoms and bonds currently in the MASK state, respectively, and $w_{b_{ij}}$ is the bond-class weight for class *b*_*ij*_.

For training, we use the 11,000 GEOM-Drugs^44^ molecules (5 conformers each; 55,000 conformers total), precomputing *V*_*K*_ at every resolution from *K* = 1 to *K* = *M* – 1 for each conformer. At every epoch, we randomly choose 30 (*S*, *V*_*K*_) pairs per conformer, so the decoder learns to reconstruct *S* from a COMPRESS representation of arbitrary resolution. The decoder is optimized with AdamW for 100 epochs with batch size of 20 per GPU. Training is performed with distributed data parallelism (DDP)^53^ across 4 NVIDIA H200 GPUs and requires approximately 60 hours.

#### 2.5.3 Inference

At inference, only a COMPRESS representation *V* and a target atom count *M* are required. We construct *S*_0_, initialize all atom types and bonds to their MASK state, and integrate the model’s learned probability flow from *t* = 0 to *t* = 1 over a fixed number of Euler steps (*N* = 200).

At each step, continuous variables (position and charge) are updated by Euler integration of the ODE $\frac{dS}{dt}=u_{t}$ , with velocity implied by the network’s end point prediction, as shown in eqs 21 and 22:

21 $$u_{t}=\frac{{Ŝ}_{1}-S_{t}}{1-t}$$

22 $$S_{t+\Delta t}=S_{t}+u_{t}\Delta t$$

Discrete variables (atom type, bond order) are updated with a continuous-time Markov chain (CTMC) sampling scheme following Campbell et al.^51^ The rate matrix $R^{i}\left(A_{t}^{i},\;j\right)$ is defined in eq 23,

23 $$R^{i}\left(A_{t}^{i},\;j\right)=\frac{1+\eta t}{1-t}p_{1|t}^{i}\left(j|A_{t}\right)\delta \left(A_{t}^{i},\;M\right)+\eta \left(1-\delta \left(A_{t}^{i},\;M\right)\right)\delta \left(j,\;M\right)$$

where *M* denotes the MASK token, $A_{t}^{i}$ is atom *i*’s current discrete state at time *t*, and $p_{1|t}^{i}\left(j|A_{t}\right)$ is the model’s predicted probability that atom *i*’s final state is class *j*. This rate matrix implies that a currently masked token is revealed, to a class sampled from $p_{1|t}^{i}$ , with probability in eq 24,

24 $$p_{\mathrm{unmask}}\left(t\right)=\Delta t\frac{1+\eta t}{1-t}$$

while an already-revealed token is returned to the masked state with probability *p*_remask_(*t*) = Δ*t η*, with η = 90.0 and Δ*t* = 1/*N* = 0.005. Both *p*_unmask_(*t*) and *p*_remask_(*t*) are clipped to [0, 1] before being used, since the raw values can exceed 1 as *t* → 1.

#### 2.5.4 Evaluation

To evaluate decoder-generated structures, we use 1,000 GEOM-Drugs molecules that were not included in training. For each molecule, we compute *V*_*K*_ at every resolution from *K* = 1 to *K* = *M* – 1 and decode each representation into a molecule using the predicted atom types, positions, and bond-order matrix. Each generated molecule is classified as failed or valid according to whether this molecule passes RDKit sanitization.^34^ We classify each valid molecule into one of three categories. First, we compute the Tanimoto similarity between Morgan (ECFP4) fingerprints of the generated molecule and the original all-atom reference: molecules with similarity 1.0 are classified as valid (reconstructed) indicating exact recovery of the input molecule. Among the remaining molecules with similarity below 1.0, we assess connectivity using RDKit (Chem.GetMolFrags) and classify them as nonfragmented or fragmented according to whether the molecule consists of more than one disconnected component. We additionally exclude 48 molecules for which the all-atom reference itself failed this construction step, retaining 37,437 (molecule, *K*) pairs across the 1,000 held-out molecules for analysis.

## 3 Results

### 3.1 Field Preservation in COMPRESS for Small Molecules

To assess how well COMPRESS preserves spatially distributed molecular fields, we first analyze representations of small molecules with varying numbers of sites, *K*. Preservation is measured using the normalized grid loss in eq 10, which compares reduced and all-atom (AA) physical fields. Equivalently, eq 10 can be written as

25 $$L_{\mathrm{norm}}\left(G_{f}^{S},\;G_{f}^{V}\right)=1-\frac{2\left\langle G_{f}^{S},\;G_{f}^{V}\right\rangle }{{∥G_{f}^{S}∥}_{2}^{2}+{∥G_{f}^{V}∥}_{2}^{2}}$$

Here, $G_{f}^{S}$ represents grid values calculated from the AA reference, while $G_{f}^{V}$ corresponds to the COMPRESS-derived grid points. As eq 25 shows, the normalized loss ranges from 0 to 2, where a value of 0 reflects perfect agreement between the two fields $\left(G_{f}^{S}=G_{f}^{V}\right)$ , 1 corresponds to orthogonality $\left(G_{f}^{S}\cdot G_{f}^{V}=0\right)$ , and 2 indicates that the fields are in opposite directions $\left(G_{f}^{S}=-G_{f}^{V}\right)$ . Thus, lower loss values indicate that the COMPRESS representation more accurately preserves the underlying physical fields.

Benzene is first used as a minimal reference system to assess how preservation changes as *K* varies from 12 down to 1. Figure 2 shows snapshots of the COMPRESS representation of a benzene molecule at different *K*. Each site is colored by partial charge (red: negative; blue: positive) and scaled by its Lennard-Jones (LJ) radius (σ). The corresponding normalized grid loss as a function of *K* is presented in Figure 3. The density, electrostatic, and van der Waals (vdW) losses increase as *K* decreases, although they show different patterns. The density loss monotonically increases, and vdW attraction and repulsion losses follow the same trend at a lower magnitude. A marked transition is observed in the total and positive-only electrostatic grid loss, reflecting the asymmetric charge distribution in benzene: negative charge is localized near the ring, while positive charge is distributed over the six peripheral hydrogen atoms. When *K* drops below 5, the COMPRESS representation can no longer maintain these distinct positive features, resulting in a sharp rise in the positive electrostatic grid loss. This behavior causes marked increases in average grid loss between *K* = 5 and *K* = 4.

**Figure 2:**
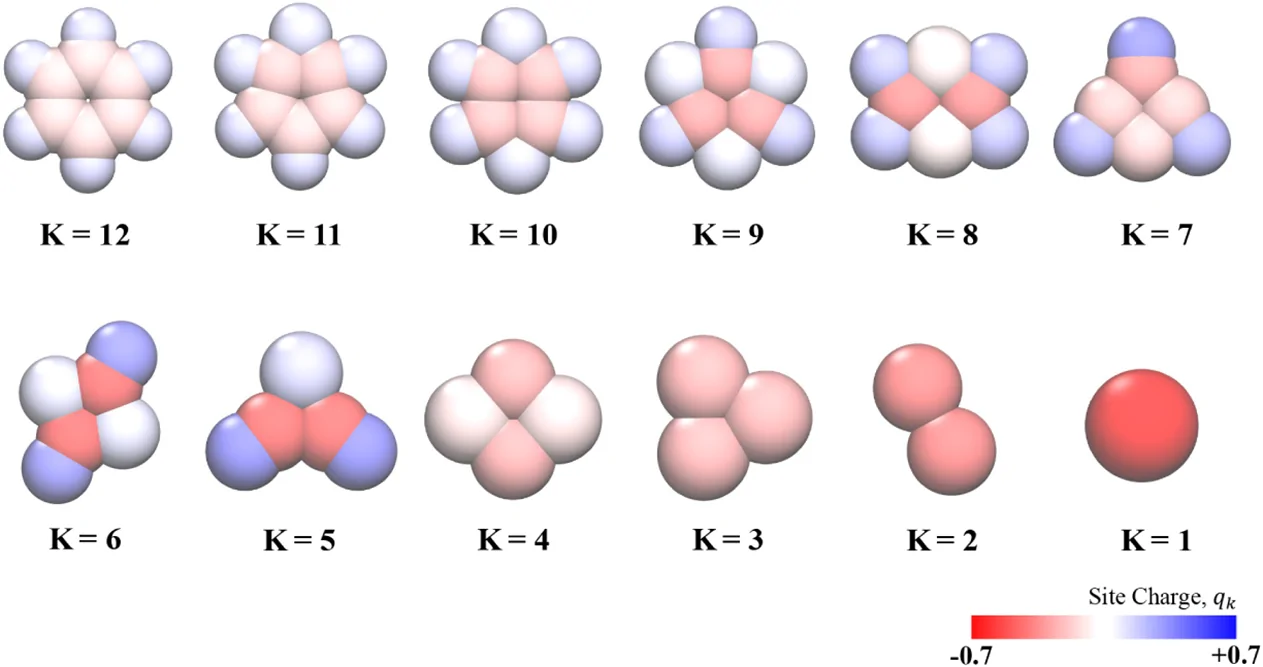
Snapshots of COMPRESS representation of a benzene molecule at different the number of sites, *K*. Each site is colored according to charge parameter of COMPRESS representation (red: negative; blue: positive) and scaled by its Lennard-Jones radius (σ). The negative sites in benzene correspond to the carbon atoms, whereas the positive sites correspond to the hydrogen atoms.

**Figure 3:**
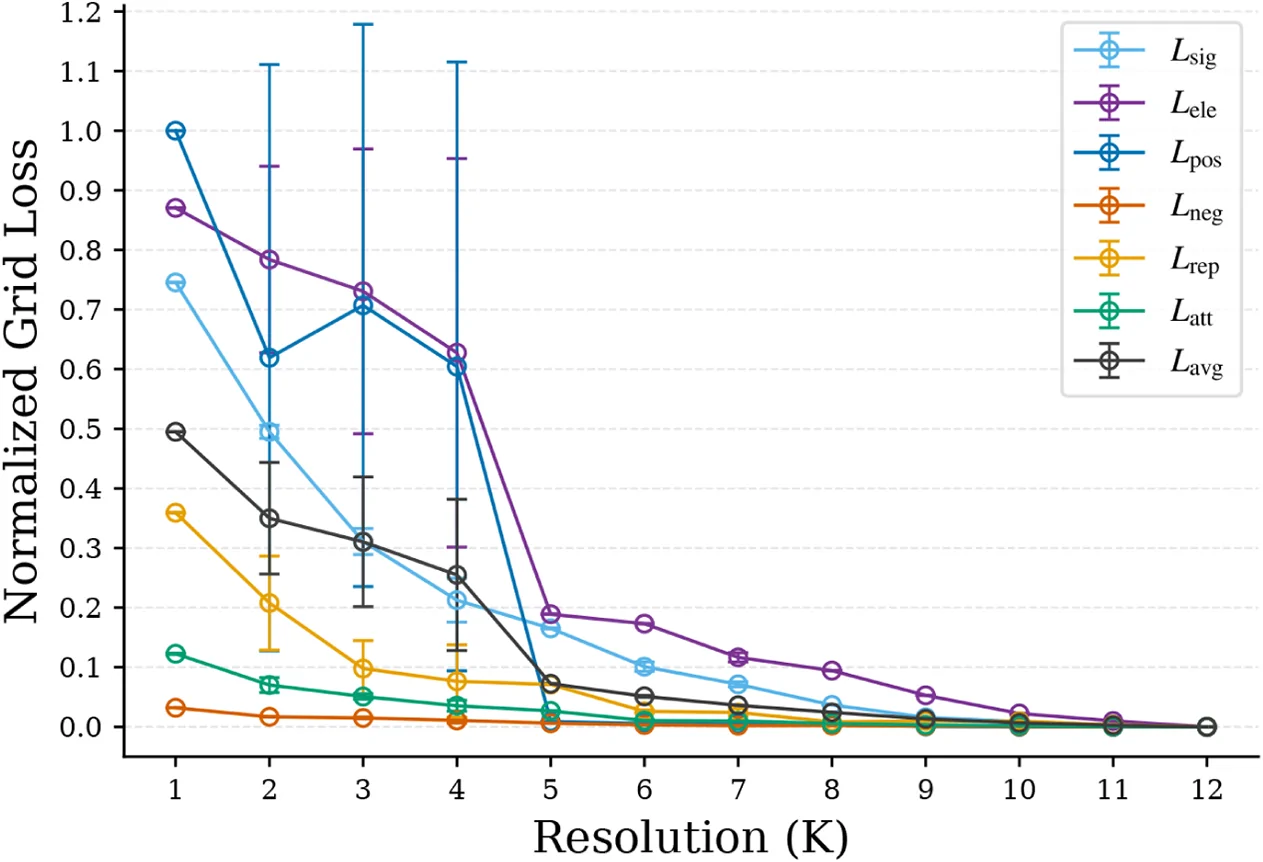
Normalized grid loss for COMPRESS representations of a benzene molecule as a function of *K*. The average loss, *L*_avg_ represents the mean of the six loss components (*L*_sig_, *L*_ele_, *L*_pos_, *L*_neg_, *L*_rep_, and *L*_att_).

The large variability in grid loss observed at lower *K* is due to variability in the optimized structures. Figure S5 shows multiple structures obtained in a set of 10 runs at each *K* value. For *K* ≥ 8 the representations shown in Figure 2 are obtained robustly. For *K* = 6 and 7, multiple representations are obtained, but with similar grid loss components. The representations obtained for *K* = 2, 3, and 4 show significant variability, which is also reflected in the grid loss components. Interestingly, larger variations are observed in the electrostatic grid loss components than are observed for the average grid loss, which shows that the different optimized representations make different trade-offs in their attempts to minimize the total loss.

We then extend our analysis to a set of 20 chemically diverse scaffold molecules. For each molecule, we plot the normalized grid loss as a function of the resolution ratio *K*/*M*, where *M* is the number of atoms in the AA molecule (Figure 4). The average grid loss is calculated as the mean of six components: *L*_sig_, *L*_ele_, *L*_pos_, *L*_neg_, *L*_rep_, and *L*_att_, as above. For clarity, we also report the average vdW grid loss as *L*_vdW_ = (*L*_rep_ + *L*_att_)/2. Although the magnitude of average grid loss differs across molecules, the overall pattern is consistent. Most systems exhibit two distinct patterns: a very slow increase in average grid loss at higher-resolution ratios, and a pronounced increase beginning in the lower-resolution transition region (0.25 < *K*/*M* < 0.5). Each component also shows similar behavior in the lowest-resolution range (*K*/*M* < 0.25), with losses approaching 1.0, indicating a complete lack of correlation. For the density loss (*L*_sig_), it increases steadily across all systems, with its slope often becoming steeper near the transition region (e.g., S15 and S16). Electrostatic loss exhibits two main behaviors: in some molecules (e.g., S7, S9, S17), it remains relatively stable until the transition region, then rises sharply, whereas in others (e.g., S3, S5 and S6), it increases with density loss starting from earlier stages of compression. This is better visualized when zoomed in on lower grid losses, as shown in Figure S2. Interestingly, some molecules with the most sensitive electrostatic grid losses are nonpolar (e.g., S6, S13). This is due to the specific form of the electrostatic loss, which divides by the magnitude of the grid elements. The vdW loss generally varies more gradually and begins to increase in the lower-resolution transition region (e.g., S1 and S13). Taken together, these results show that COMPRESS representation follows a consistent transition pattern for diverse small molecules, even though the absolute values of each component vary by molecule.

**Figure 4:**
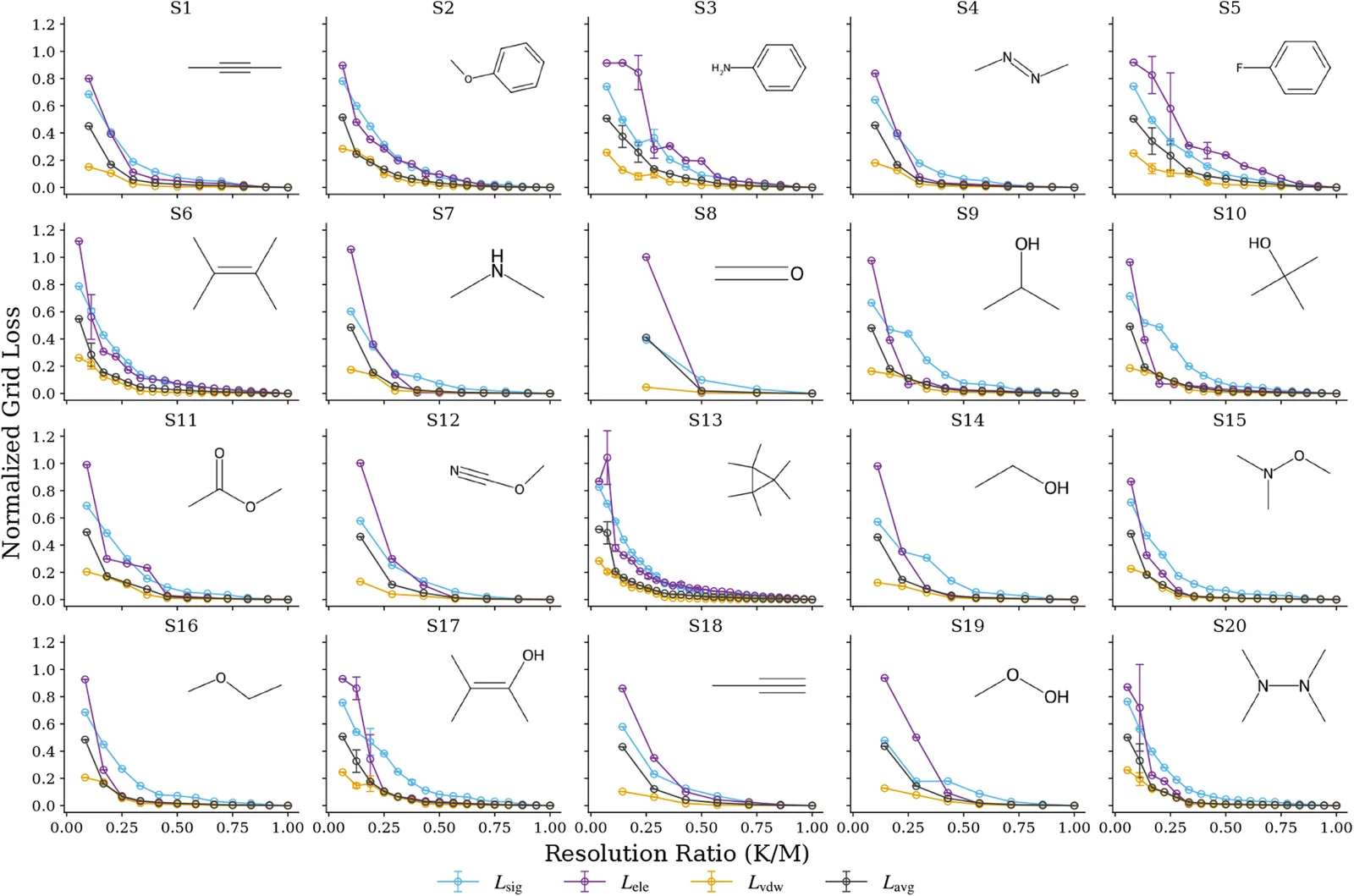
Normalized grid loss across 20 scaffold molecules as a function of resolution ratio, *K*/*M*. Each figure corresponds to a single molecular system, with the molecular structure displayed as an inset. The average grid loss, *L*_avg_ was calculated as the mean of six components: *L*_sig_, *L*_ele_, *L*_pos_, *L*_neg_, *L*_rep_, and *L*_att_. The vdW grid loss, *L*_vdW_, is defined as (*L*_rep_ + *L*_att_)/2.

### 3.2 Preservation of Intermolecular Interaction Energies

We subsequently evaluate to what extent the COMPRESS representation preserves intermolecular interaction energies relative to the AA reference. Two representative systems are examined: benzene dimers and protein–ligand complexes. For each system, we compute the electrostatic, vdW, and total interaction energies from the COMPRESS representation and examined their dependence on *K*. We construct three standard benzene dimer geometries commonly used as reference configurations (Figure 5a): face-to-face (FF), offset stacking (OFF), and face-to-edge (FE).^54,55^ The upper benzene, depicted in cyan, remains in its all-atom form, whereas only the lower benzene, shown in pink, is reconstructed with varying *K*. The corresponding total (*E*_total_), Lennard-Jones (*E*_LJ_), and Coulomb (*E*_Coulomb_) interaction energies for each geometry as functions of *K* are shown in Figure 5b,c,d, with dashed lines indicating AA reference values. Across all three geometries, at high resolution (*K* ≥ 5), changes in the total energy are primarily driven by changes in *E*_LJ_, which increases smoothly as *K* decreases. In contrast, the Coulomb term is relatively flat. The Coulomb term shows positive values in FF (approximately 2.24 kcal/mol) and OFF (approximately 1.18 kcal/mol), indicating weak electrostatic repulsion, and a slightly negative value in FE (approximately −0.77 kcal/mol), indicating weak attraction. For *K* < 5, *E*_Coulomb_ changes dramatically and becomes main contributor to differences in *E*_total_, becoming more repulsive in FF and OFF, while becoming more attractive in FE. To elucidate the observed changes in *E*_Coulomb_, we calculate the Coulomb interaction energy for each atom in an AA benzene molecule with the COMPRESS representation across all three geometries (Table 2). *E*_*C*_ denotes the mean Coulomb interaction energy for the six carbon atoms in the AA benzene with the COMPRESS representation, while *E*_*H*_ represents the corresponding mean for the six hydrogen atoms. Across all geometries, both *E*_*C*_ and *E*_*H*_ remain stable for *K* ≥ 5, but exhibit significant changes for *K* < 5: *E*_*C*_ shifts to more positive values, while *E*_*H*_ becomes more negative. This more clearly shows the deterioration of the electrostatics, as the differences in *E*_*C*_ and *E*_*H*_ are much larger than the net changes, due to a cancellation of errors. This observation suggests that *K* = 5 is the minimum number of sites required to preserve benzene’s electrostatic pattern in these geometries. This transition is further supported by the grid loss analysis of a single benzene molecule (Figure 3), which indicates that the same resolution limit governs the grid loss.

**Table 2:** Mean Coulomb Interaction Energies per Carbon (*E_C_*; Averaged over Six Carbon Atoms) and per Hydrogen (*E_H_*; Averaged over Six Hydrogen Atoms) on the AA Benzene Interacting with the COMPRESS Representation, Shown for FF, OFF, and FE Geometries as a Function of the *K*

	FF (kcal/mol)		OFF (kcal/mol)		FE (kcal/mol)	
*K*	*E*_*C*_	*E*_*H*_	*E*_*C*_	*E*_*H*_	*E*_*C*_	*E*_*H*_
1	7.14	–6.34	6.61	–6.06	5.51	–5.79
2	4.96	–4.39	4.44	–4.11	3.82	–4.02
3	5.31	–4.72	4.94	–4.51	4.10	–4.30
4	4.68	–4.11	4.33	–3.93	3.59	–3.79
5	0.83	–0.50	0.66	–0.53	0.52	–0.65
6	0.83	–0.50	0.64	–0.47	0.52	–0.65
7	0.85	–0.49	0.66	–0.46	0.52	–0.65
8	0.85	–0.48	0.62	–0.44	0.51	–0.63
9	0.82	–0.46	0.61	–0.43	0.50	–0.63
10	0.84	–0.46	0.62	–0.43	0.50	–0.63
11	0.84	–0.47	0.63	–0.43	0.50	–0.63
12	0.84	–0.47	0.63	–0.43	0.50	–0.63

**Figure 5:**
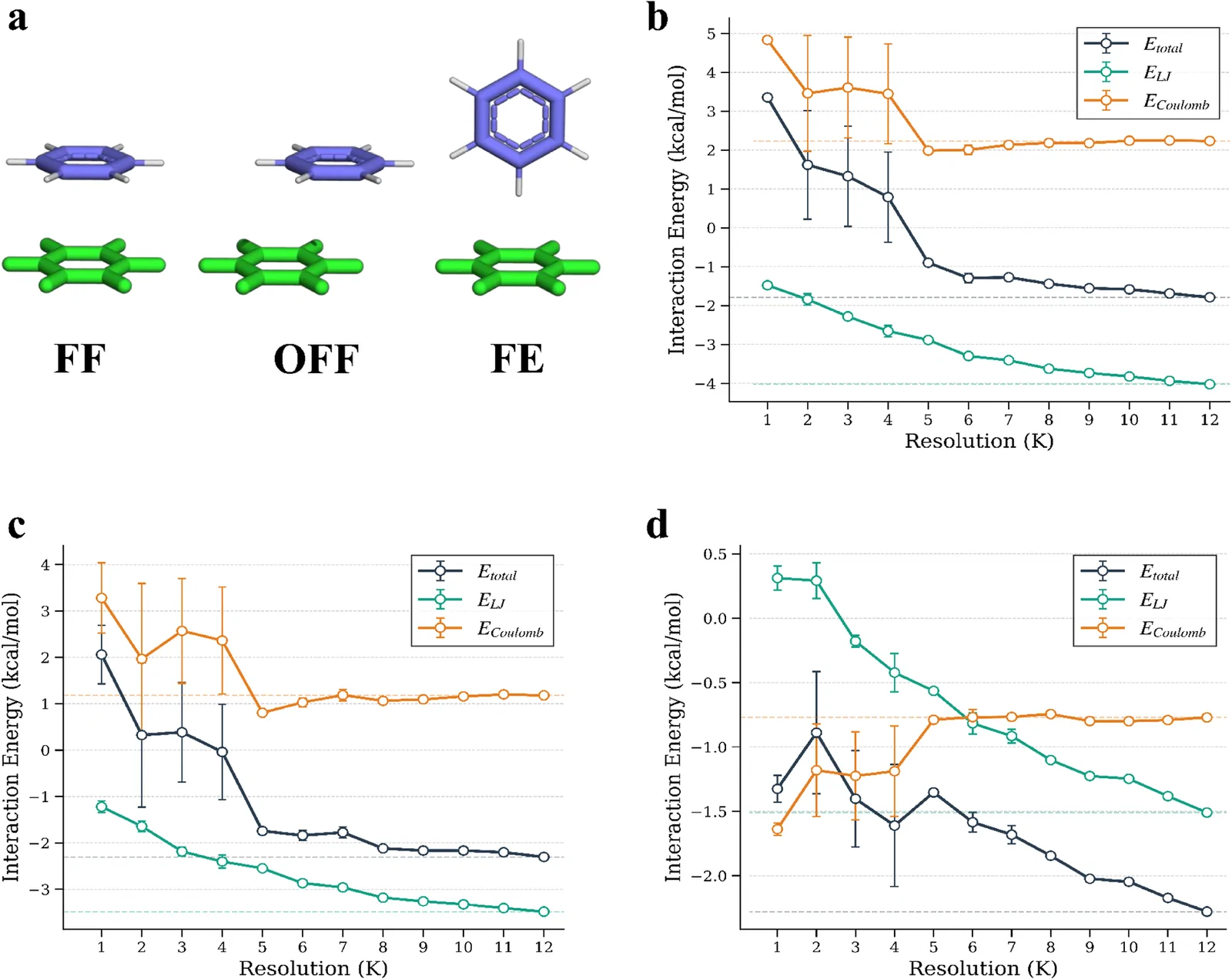
Structures of three benzene dimer geometries (a): face-to-face (FF), offset (OFF), and face-to-edge (FE). The upper benzene (purple) remains in its all-atom form, whereas the lower benzene (green) is represented using COMPRESS. Corresponding Coulomb (*E*_Coulomb_), Lennard-Jones (*E*_LJ_), and total (*E*_total_) interaction energies are shown in (b), (c), and (d), respectively, as functions of *K*. Dashed horizontal lines indicate the corresponding all-atom reference values.

We extend our analysis to two protein–ligand complexes: trypsin (PDB: 3PTB) and the β_2_-adrenergic receptor (B2AR) (PDB: 2RH1), bound to the ligands benzamidine and carazolol, respectively. Figure 6a,b shows the ligand structures of benzamidine and carazolol, while Figure 6c,d presents the corresponding interaction energies of the COMPRESS representation with the protein at various *K*, decomposed into *E*_total_, *E*_LJ_, and *E*_Coulomb_. Dashed lines indicate the AA reference values. Both systems exhibit a qualitative pattern similar to that observed with the benzene dimers. As *K* decreases, *E*_LJ_ increases monotonically, while *E*_Coulomb_ remains relatively stable until the resolution reaches a system-specific transition: *K* = 7 in 3PTB and *K* ≈ 24–26 in 2RH1. Figure 7 shows the protein–ligand interaction energy error $\left|\Delta E\left(K\right)\right|=\left|E_{\mathrm{total}}^{\mathrm{COMPRESS}}\left(K\right)-E_{\mathrm{total}}^{\mathrm{AA}}\right|$ as a function of the average grid loss across *K*. At the lowest grid losses, |Δ*E*(*K*)| remains close to zero, but grows with grid loss. Vertical dashed lines mark average grid losses of 0.02 and 0.05, indicating the approximate range beyond which |Δ*E*(*K*)| begins to rise sharply, with the annotated points; *K* = 6 for trypsin and *K* = 23 for B2AR. This suggests that degradation of grid field agreement leads to a rapid increase in interaction energy error with the protein. We further examine other molecular properties as a function of grid loss (Figure S3). The dipole magnitude and cosine similarity of the dipole direction exhibit trends consistent with those observed for the interaction energies. In contrast, the radius of gyration (*R*_*g*_) shows reduced sensitivity, with no significant deterioration until grid losses exceed 0.20 to 0.30.

**Figure 6:**
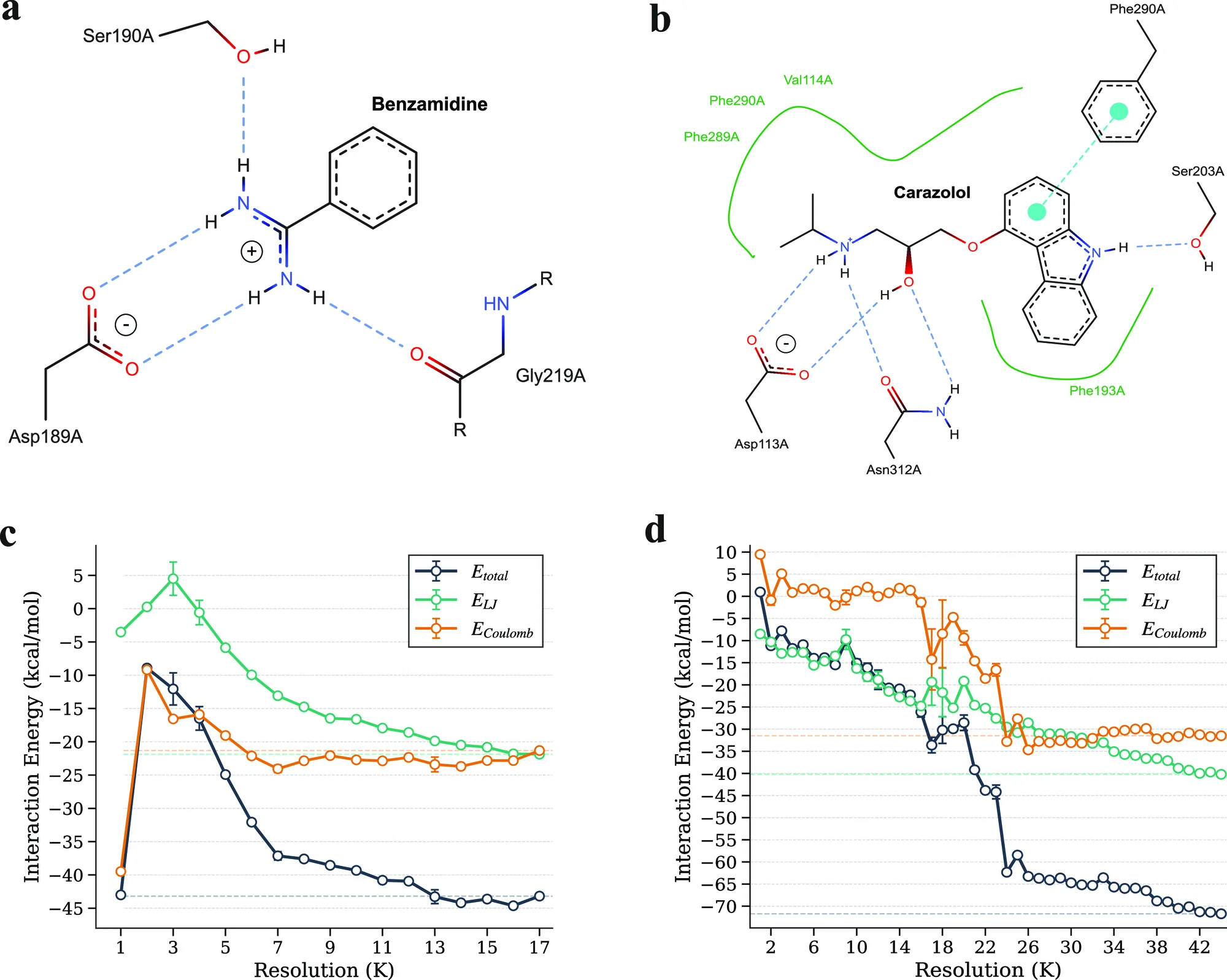
Ligand structures and representative interactions for two protein–ligand complexes: (a) benzamidine bound to trypsin (PDB: 3PTB), and (b) carazolol bound to B2AR (PDB: 2RH1). Poseview diagrams^56^ were constructed using the ProteinsPlus Web server.^57^ Corresponding Coulomb (*E*_Coulomb_), Lennard-Jones (*E*_LJ_), and total (*E*_total_) interaction energies between the protein and the COMPRESS representation of the ligand are shown in (c) and (d) for trypsin and B2AR, respectively, as functions of *K*. Dashed horizontal lines indicate the corresponding all-atom reference interaction energies.

**Figure 7:**
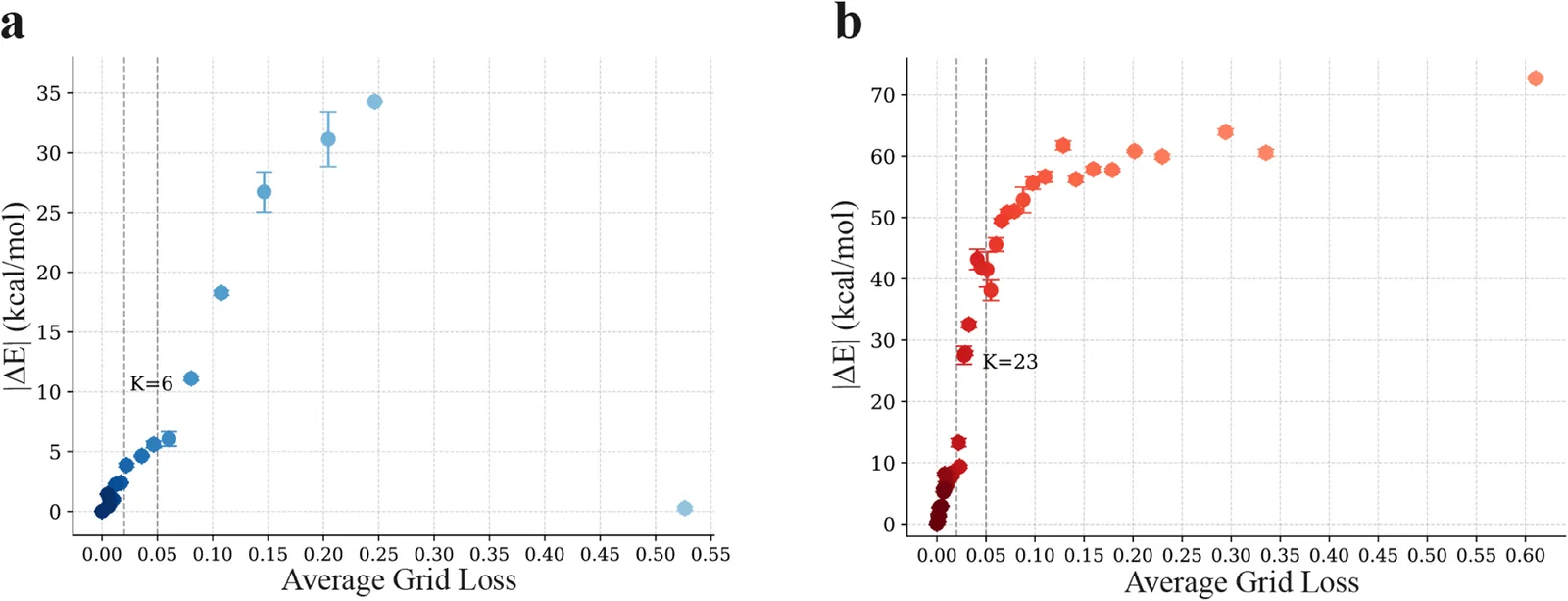
Relationship between average grid loss and the absolute error of the protein–ligand interaction-energy |Δ*E*(*K*)| = |*E*(*K*)_COMPRESS_ – *E*_AA_|, across different *K* for (a) trypsin and (b) B2AR. Darker colors indicate larger *K*. Vertical dashed lines indicate average grid losses of 0.02 and 0.05, marking the approximate range beyond which |Δ*E*(*K*)| begins to rise sharply; annotated points (*K* = 6 for trypsin and *K* = 23 for B2AR) mark specific *K* values near this range.

We next compute residue-wise interaction energy deviations between the COMPRESS representation and the AA ligand for trypsin and B2AR (Figure 8). We first select 30 key residues which have the largest total interaction energies with the AA ligand (benzamidine for trypsin and carazolol for B2AR) for each complex, then compute residue-wise deviations for the Coulomb (Δ*E*_Coulomb_) and LJ (Δ*E*_LJ_) interaction energies. For Δ*E*_Coulomb_, the largest changes are concentrated in the top-ranked electrostatic residues: ASP189 in 3PTB, and ASP113 in 2RH1. For both amino acids, Δ*E*_Coulomb_ is near zero at high resolution, drifts negative as *K* decreases, and then increases sharply once *K* enters the low-resolution range. Deviations in other residues are present but smaller in magnitude, indicating that the Coulomb energy change in Figure 6 is dominated by the top-ranked residue in each complex. For Δ*E*_LJ_, the deviation is not dominated by a single residue; instead, most residues show a gradual change as *K* decreases, to a lesser extent than Δ*E*_Coulomb_. In 2RH1, ASP113 exhibits the largest Δ*E*_LJ_ magnitude compared with the other residues, but the effect is still much less concentrated than the ASP113-driven behavior observed for Δ*E*_Coulomb_.

**Figure 8:**
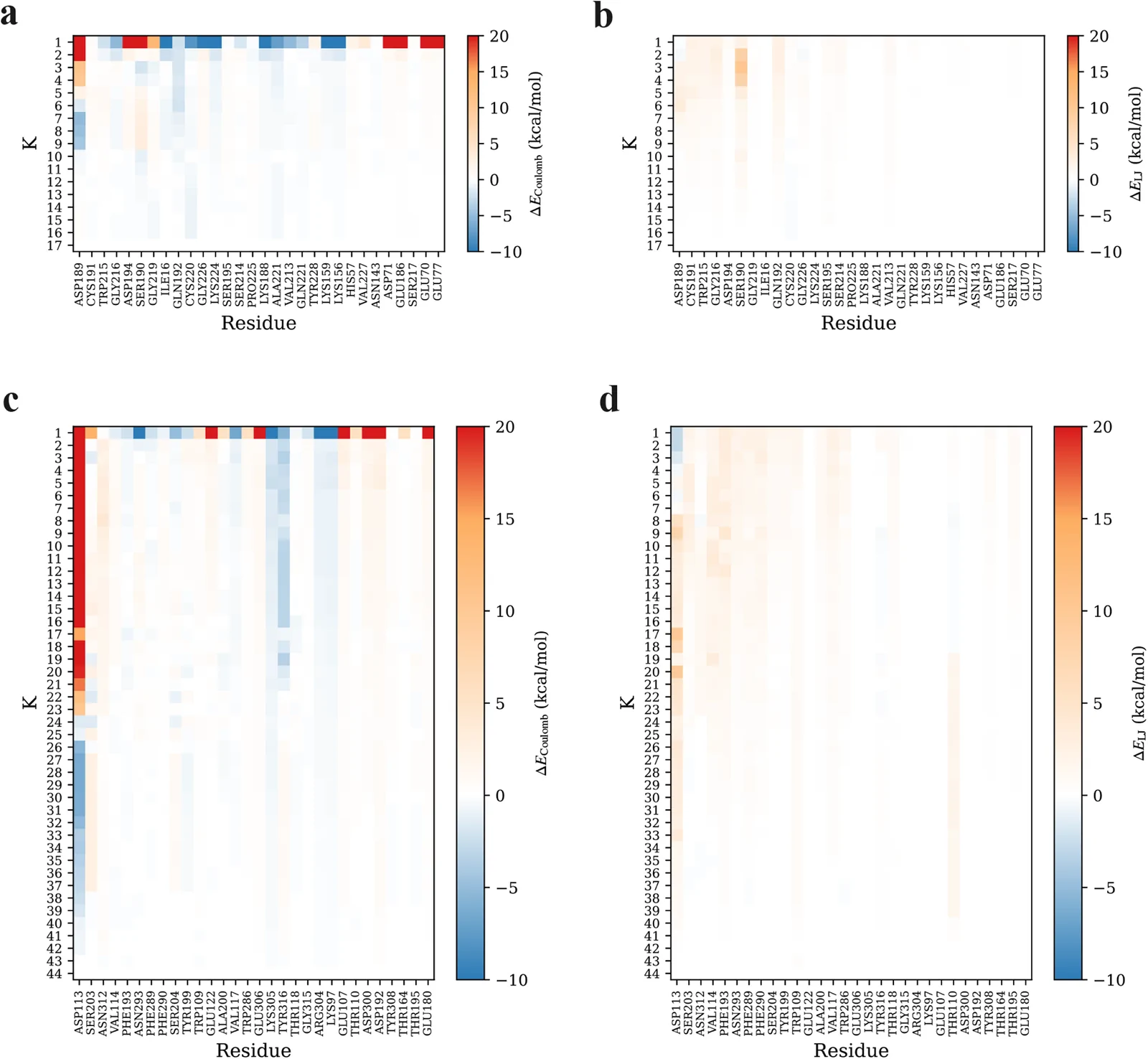
Heatmaps of residue-wise interaction-energy deviations between the COMPRESS representation and the all-atom ligand for the 30 key residues in each complex as a function of *K*. (a) Coulomb (Δ*E*_Coulomb_) and (b) Lennard-Jones (Δ*E*_LJ_) deviations for the trypsin complex (PDB: 3PTB). (c) Coulomb (Δ*E*_Coulomb_) and (d) Lennard-Jones (Δ*E*_LJ_) deviations for the B2AR complex (PDB: 2RH1). Residues are ordered by the magnitude of their total interaction energy with the all-atom ligand in each complex.

Collectively, we find that both Coulomb and LJ interactions deteriorate as resolution decreases in the three benzene dimer geometries and both protein–ligand complexes. The LJ interaction increases gradually, whereas the Coulomb interaction exhibits a more pronounced, resolution-dependent transition, leading to a rapid escalation in the total interaction energy error. Also, the collapse of grid field preservation is predictive of failures to capture interaction energies and other global molecular properties, suggesting a practical guideline for resolution selection: an average grid loss below approximately 0.05 is needed for accurate retention of molecular properties in COMPRESS representations.

### 3.3 Large-Scale Statistical Analysis across Drug-like Molecules

We next perform a statistical analysis to evaluate the performance of COMPRESS across a larger and more chemically diverse data set. COMPRESS representations are generated for 10,000 molecules (five conformers per molecule, 50,000 conformers in total) from the GEOM-Drugs data set^44^ at *K* = 10, 15, 20, and 25. Representative examples are shown in Figure 9, which contrasts AA molecules with their COMPRESS representations at different *K*, annotated with the number of atoms *M* and the corresponding average grid loss *L*_avg_ for each COMPRESS representation. For the all representatives, AA molecules are reconstructed with complete accuracy when *K* = *M*. As the value of *K* decreases, the reconstruction error increases. Additionally, for a fixed *K*, the average loss is lower for molecules with smaller *M* and increases as *M* increases. These results indicate that molecular preservation depends on both the absolute resolution *K* and the resolution ratio *K*/*M*.

**Figure 9:**
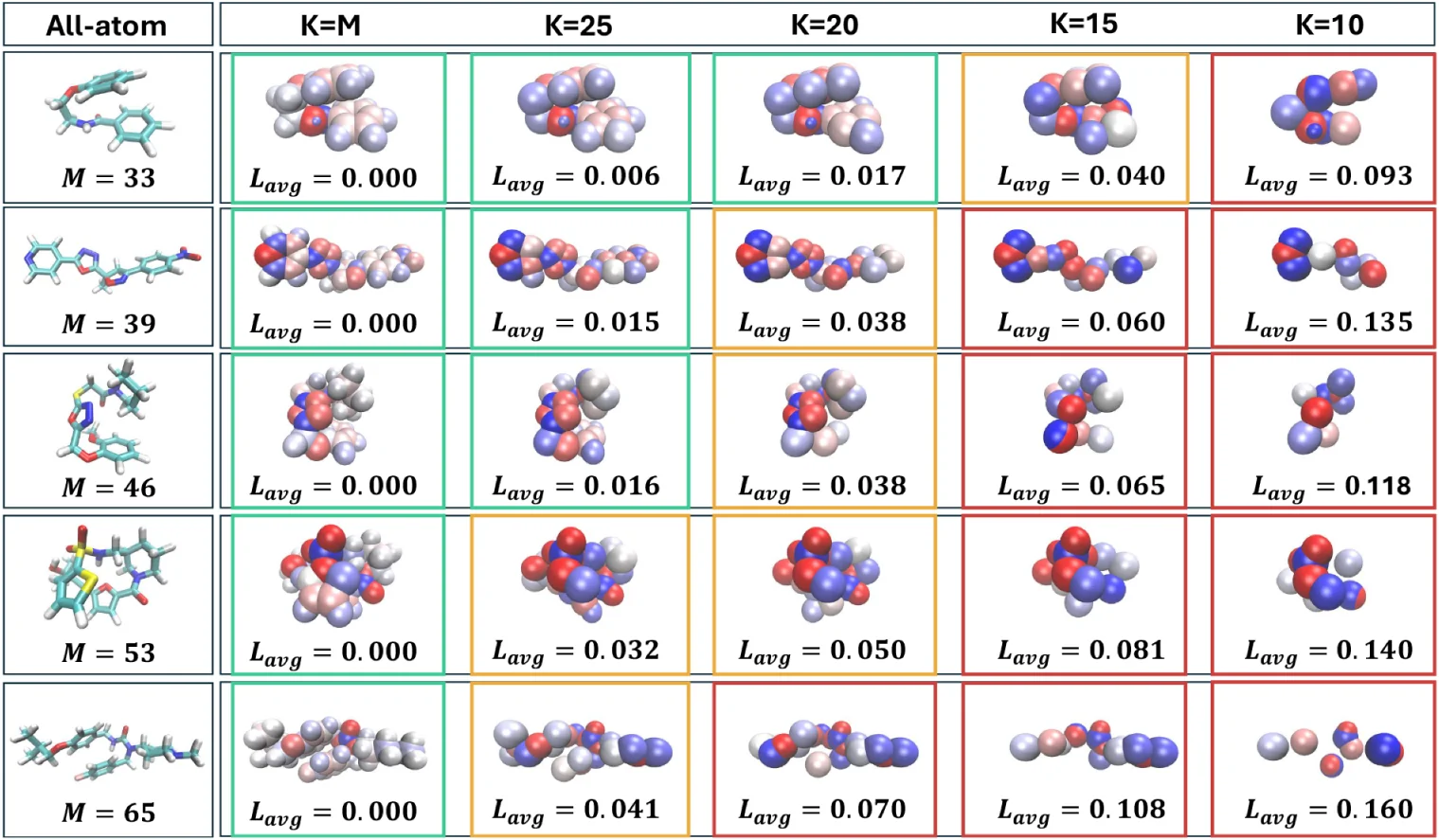
Snapshots of COMPRESS representations for representative molecules at *K* = 10, 15, 20, 25, and *M*. The leftmost column shows the corresponding all-atom structures. Sites are colored by charge parameter (red: negative; blue: positive) and scaled by their Lennard-Jones radius parameter (σ). *M* denotes the number of atoms in the AA structure, and *L*_avg_ is the average grid loss of the COMPRESS representation. Box colors indicate the average grid loss range: green (*L*_avg_ < 0.02), orange (0.02 ≤ *L*_avg_ < 0.05), and red (*L*_avg_ ≥ 0.05).

Figure 10 shows the average grid loss distributions across 50,000 conformers at *K* = 10, 15, 20, and 25. As *K* decreases, the distributions shift toward higher loss and exhibit a broader distribution. The three vertical reference lines at 0.02, 0.05, and 0.10 serve to summarize performance of COMPRESS at a fixed *K*. The choice of cutoffs is guided by the protein–ligand complex results in Figure 7, where the absolute interaction energy error begins to rise at an average grid loss around 0.02–0.05. The corresponding fractions of structures below each cutoff are summarized in Table 3. The fraction of conformers with grid loss below 0.05 is 96.90% at *K* = 25 and 70.69% at *K* = 20, but decreases sharply to 17.69% at *K* = 15 and 1.08% at *K* = 10 This indicates representations with low *K* rarely remain in the low-loss range (<0.05). Therefore, reducing *K* shifts the data set toward higher average grid loss and weakens agreement with the molecular fields of AA molecules, leading to significant performance deterioration when *K* ≤ 15.

**Table 3:** Percentages of the 50,000 GEOM-Drugs Conformers Falling below Cumulative Average Grid-Loss Cutoffs at Different *K*

*K*	<0.02	<0.05	<0.1	≥0.1
10	0.00	1.08	15.69	84.31
15	1.06	17.63	87.59	12.40
20	13.92	70.69	99.86	0.15
25	51.36	96.90	100.00	0.00

**Figure 10:**
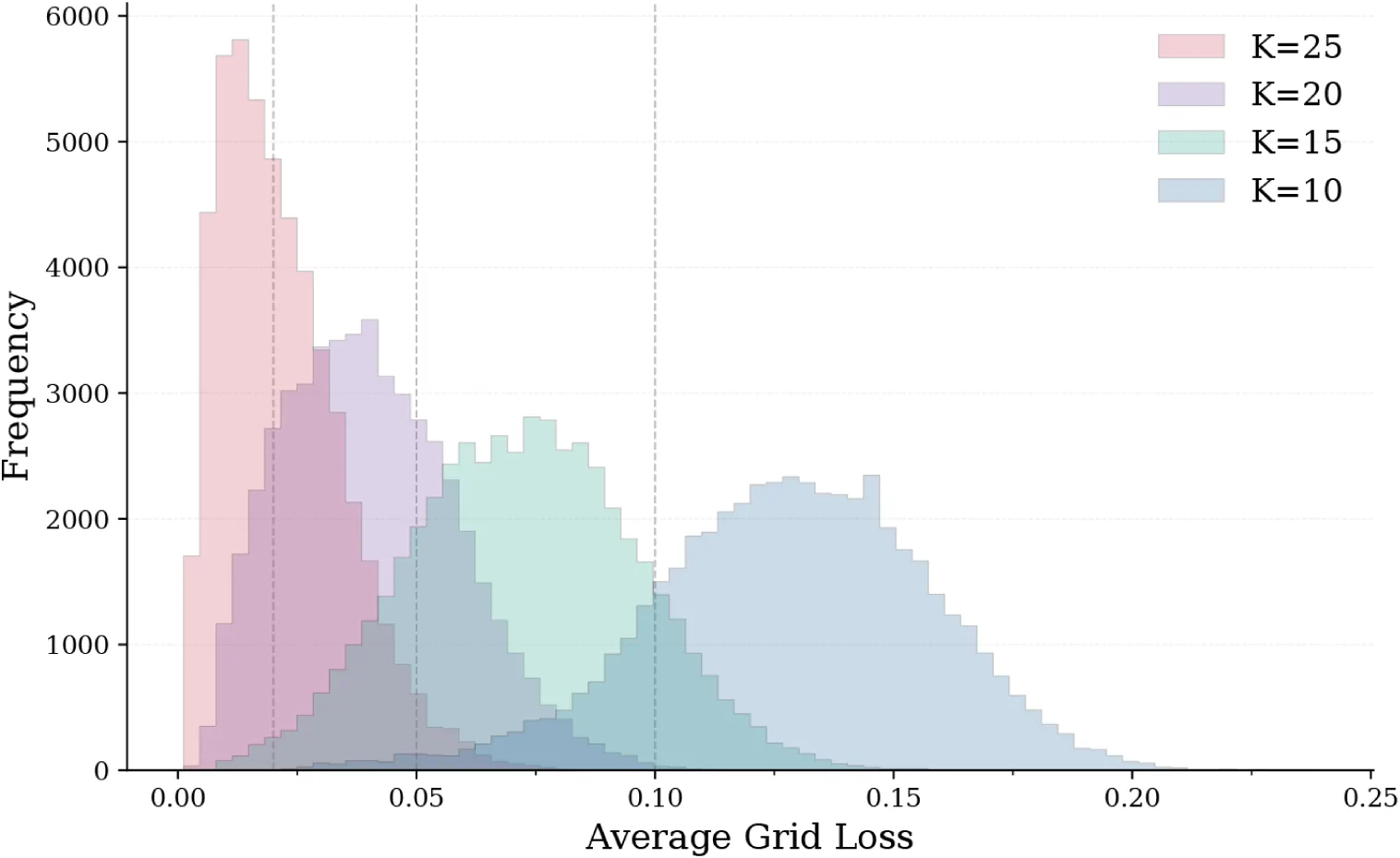
Average grid-loss distributions for 50,000 GEOM-Drugs conformers represented by COMPRESS at *K* = 10, 15, 20, and 25. Vertical dashed lines at 0.02, 0.05, and 0.10 indicate reference loss levels used as practical guidelines for comparing the fraction of conformers below each value across *K*.

Two global properties—the radius of gyration (*R*_*g*_) and dipole moment (μ)—are compared between COMPRESS representations and AA references across varying values of *K* (Figure 11). The differences in properties are defined as $\Delta R_{g}=R_{g}^{\mathrm{AA}}-R_{g}^{\mathrm{COMPRESS}}$ and Δμ = μ^AA^ – μ^COMPRESS^, and the dipole direction agreement is quantified by the cosine similarity cos θ_μ_ = ***μ̂*** ^COMPRESS^ · ***μ̂*** ^AA^. The *R*_*g*_ results are summarized in Figure 11a,d–g. As *K* decreases, Δ*R*_*g*_ shifts toward larger positive values and the distribution widens. This trend indicates underestimation of *R*_*g*_ at lower *K* (Figure 11a). The scatter plots (Figure 11d–g) demonstrate that the Pearson correlation decreases as *K* drops (R = 0.944 at *K* = 25, 0.912 at *K* = 20, 0.830 at *K* = 15, and 0.688 at *K* = 10). A notable drop occurs between *K* = 20 and *K* = 15. For the dipole moment μ, both magnitude and direction are compared in Figure 11b,c,h–k. As *K* decreases, Δμ increases and its distribution broadens, similar to the behavior observed for Δ*R*_*g*_. The cosine similarity also decreases, indicating that both the magnitude and direction of μ increasingly deviate from the AA reference at lower resolutions. The Pearson correlation for the dipole moment (Figure 11h–k) deteriorates as *K* decreases, but remains higher than that observed for *R*_*g*_ (R = 0.985 at *K* = 25, 0.970 at *K* = 20, 0.943 at *K* = 15, and 0.893 at *K* = 10). These large-scale analyses show that increasing *K* systematically improves the COMPRESS representation: the fraction of conformers below *L*_avg_ = 0.05 grows from ∼1% at *K* = 10 to ∼97% at *K* = 25, and the recovery of *R*_*g*_ and the dipole moment improves accordingly. Together, these results identify *K* = 25 as a practical resolution for drug-like molecules, while *K* ≤ 15 leads to substantial loss.

**Figure 11:**
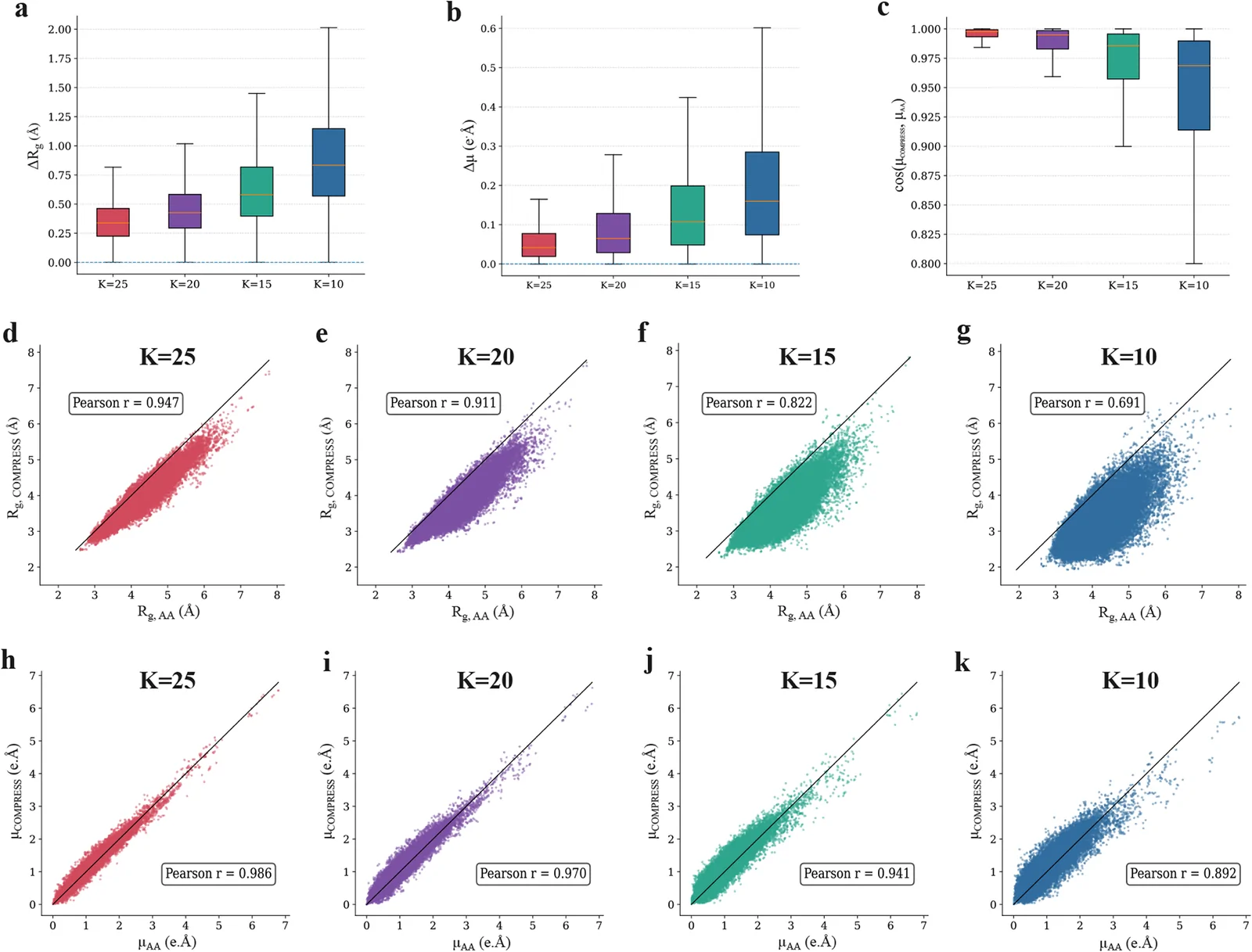
Box plots of (a) Δ*R_g_* (radius-of-gyration difference), (b) Δμ (dipole-moment difference), and (c) cosine similarity of dipole directions (cos­(μ_COMPRESS_, μ_AA_). Scatter plots compare COMPRESS and AA values of (d–g) *R_g_* and (h–k) dipole moment at different *K*.

### 3.4 COMPRESS Decoder

To evaluate whether COMPRESS representations can support molecular generation, we introduce a conditional flow-matching decoder, Ψ_θ_, that generates chemically valid all-atom structures directly from a COMPRESS representation, without reference to the original all-atom molecule (Figure 12). We assess this decoder in two ways: first, by quantifying reconstruction validity across a range of resolution ratios *K*/*M*, and second, by examining how perturbations of a COMPRESS representation yield alternative structures.

**Figure 12:**
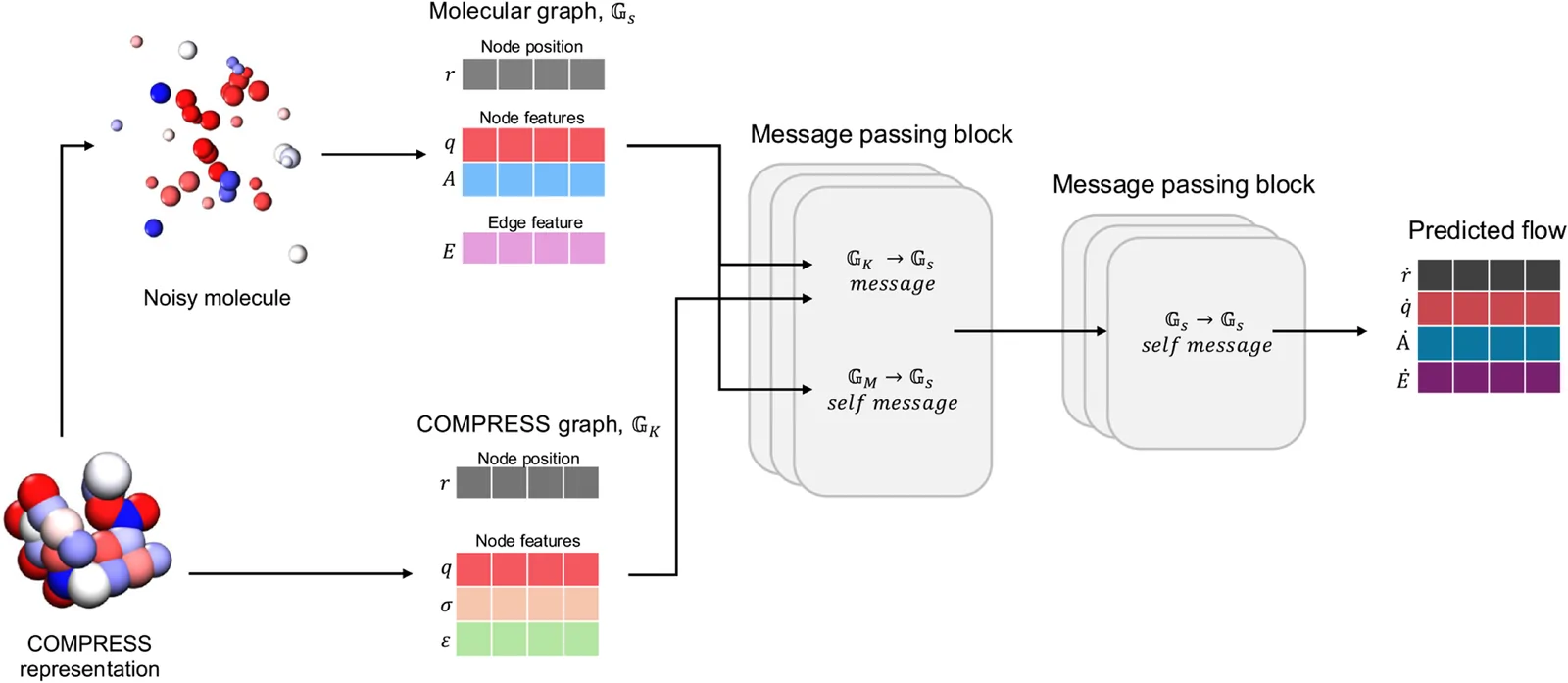
Architecture of the COMPRESS decoder Ψ_θ_. The noisy candidate structure is encoded as a graph *G_S_* and iteratively updated through message-passing blocks, conditioned on the COMPRESS representation *V*, encoded as a graph *G_V_*, to predict the molecular structure.

We decode COMPRESS representations at every resolution *K* = 1 to *M* – 1 for 1000 molecules and classify each resulting structure as failed or valid. Figure 13a shows these proportions as a function of resolution ratio *K*/*M*. The valid fraction rises from 29.7% at *K*/*M* < 0.1 to 93.1% at *K*/M = 0.9–1.0. This rise is steep only up to *K*/*M* ≈ 0.5–0.6, where the valid fraction is already 86.5%. Beyond this point it gains only 6.6 percentage points across the remaining half of the resolution range. This plateau matches the grid-loss behavior described before: COMPRESS representations preserve field agreement well up to *K*/*M* ≈ 0.5, then lose it sharply in the 0.25 < *K*/*M* < 0.5 transition region. Thus, the drop in the fraction of valid structures at low *K*/*M* likely reflects this same loss of information in the COMPRESS representation, rather than a limitation of the decoder itself.

**Figure 13:**
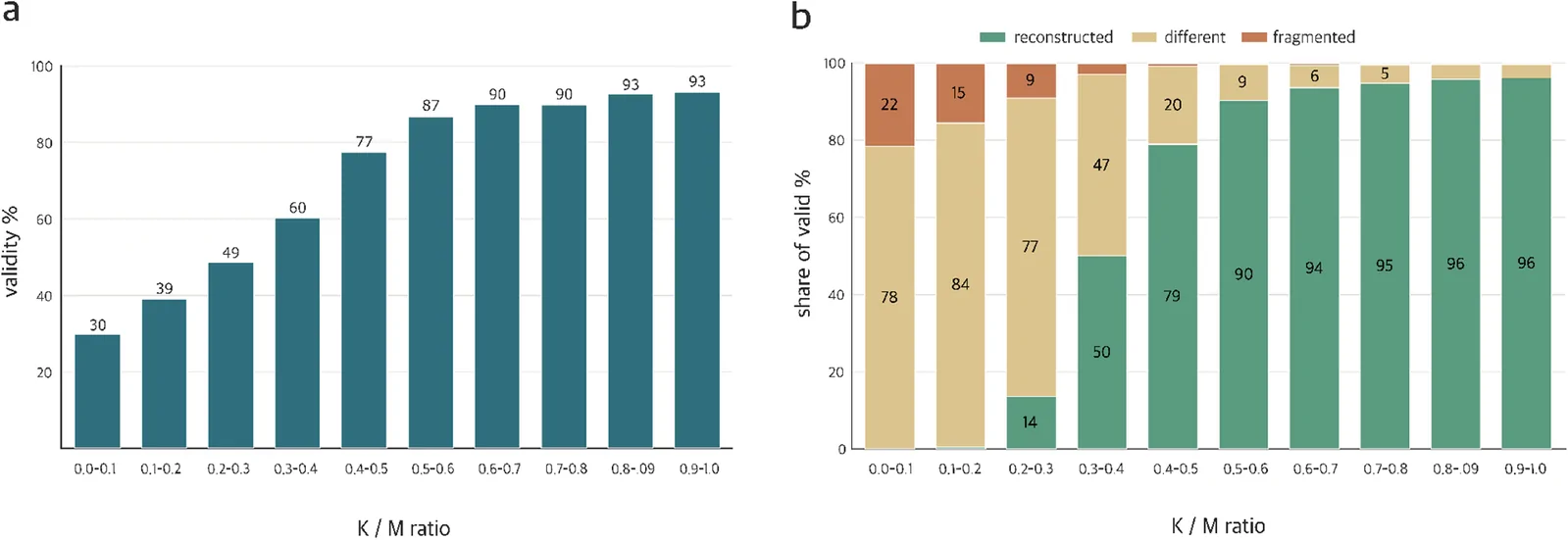
Reconstruction performance of the COMPRESS decoder across resolution ratios *K*/*M*. (a) Fraction of decoded structures that are chemically valid. (b) Among valid structures, the proportion that exactly reconstruct the reference molecule (reconstructed), are a distinct but single-component valid molecule (different), or are fragmented into multiple components (fragmented).

Figure 13b shows how valid structures break down into three outcomes: an exact match to the reference (reconstructed), a distinct but single-component valid molecule (different), fragmented valid molecule (fragmented). At the lowest resolution ratios, reconstructed structures are essentially absent (0.5% or less for 0.0 ≤ *K*/*M* ≤ 0.2), and nearly all valid structures are different or fragmented. As *K*/*M* increases, the fraction of reconstructed molecules rises sharply (50.0% by *K*/M = 0.3–0.4 and 90.4% by *K*/M = 0.5–0.6), while others decline in parallel. Beyond *K*/*M* ≈ 0.6, the composition stabilizes: reconstructed structures make up 93–96% of valid structures. Different molecules dominate the remaining valid structures at low resolution (78.4% at *K*/*M* < 0.1) and decline steadily as *K*/*M* increases, only being overtaken by exact reconstructions around *K*/*M* ≈ 0.3–0.4. Fragmentation, in contrast, is consistently the minority outcome, peaking at 21.6% at *K*/*M* < 0.1 and falling below 1% once *K*/*M* ≳ 0.4. Overall, COMPRESS representations support molecular generation across the full range of *K*/*M*: even at low resolution a meaningful fraction of generated structures are valid, and reconstruction fidelity rises steadily as *K*/*M* increases, with an exact match to the reference becoming reliable once *K*/*M* ≳ 0.4–0.5 and performance saturating by *K*/*M* ≈ 0.6–0.7.

To qualitatively examine how the decoder responds to perturbations of a COMPRESS representation, we select a single molecule, compress it to *K* = 15 sites (Figure 14a), and carefully modify individual site parameters or the site count before decoding, while also changing the target atom count *M* accordingly. We first perturb the *q* and ϵ of the rightmost site (Figure 14b,c). Making its charge more negative, while increasing its well depth (Δ*q* = −0.5, Δϵ = 0.3, ΔM = +3; Figure 14b), leads the decoder to generate two hydroxyl (−OH) groups at that site, while the rest of the molecule remains unchanged. Making it more positive instead, with the same change in well depth (Δ*q* = +0.5, Δϵ = 0.3, ΔM = +1; Figure 14c), instead places a nitrogen atom on the underside of the site, making that region of the molecule relatively more positive. We next increase the σ of the leftmost site (Δσ = +0.6, ΔM = +3; Figure 14d). This site is decoded into a three-carbon ring, increasing the size of the local structure to match its enlarged radius. Finally, we rationally remove sites (Δ*K* = −3, ΔM = −5; Figure 14e) or add sites (Δ*K* = +7, ΔM = +21; Figure 14f). Removing sites deletes the corresponding atoms while the remaining structure appears to retain its electrostatic character, whereas adding sites generates a new functional group whose pattern resembles that of the added sites. Together, these results show that the decoder follows the shape and attribute pattern of the COMPRESS representation, leaving the unperturbed parts of the molecule intact while reflecting each modification at its corresponding site. This highlights a practical advantage of using a physically interpretable representation like COMPRESS for molecular generation: the representation can potentially be adjusted with intent, guiding the decoder toward molecules with desired structural or chemical features.

**Figure 14:**
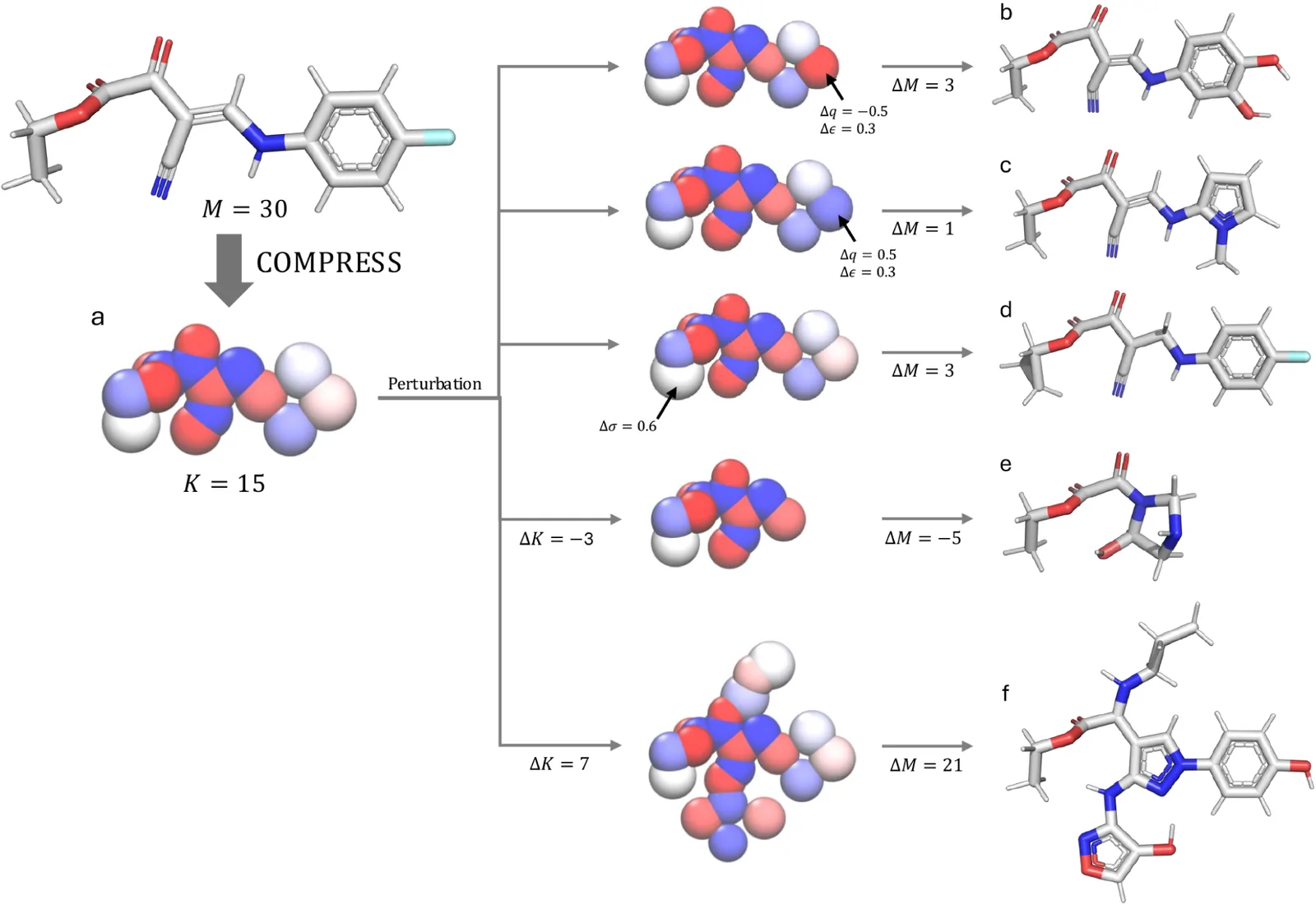
Effect of perturbing a COMPRESS representation on the decoded molecule, starting from a baseline representation (a, *K* = 15, M = 30). Perturbing a single site’s charge, ϵ, or σ (b–d) alters the corresponding local functional group in the generated molecule, while adding or removing sites (e–f) adds or removes atoms at that location.

## 4 Discussion

In this study, we present COMPRESS, an optimization framework that represents each molecule as a fixed-size set of *K* physically parametrized sites. Each site is defined by a position and three nonbonded interaction parameters: charge (*q*), Lennard-Jones (LJ) diameter (σ), and well depth (ϵ). This representation is optimized to reproduce the density, electrostatic, and van der Waals (vdW) grid fields of the corresponding all-atom molecule. By matching molecular fields across multiple smoothing scales (τ), the framework captures both global and local aspects of molecular structure. This representation therefore captures not only molecular geometry, but also spatially distributed physical information in a fixed-size, directly interpretable form of *K* × 6 parameters.

The fidelity of the representation can be measured in multiple ways. Across systems, decreasing *K* increases the density, electrostatic, and vdW grid losses: slowly at high resolution, and more rapidly within a system-dependent transition region. Corresponding degradation is also observed in intermolecular interaction energies. LJ interaction energy errors increase gradually as *K* decreases, whereas Coulomb interaction energy errors remain comparatively stable at higher resolution and then rise sharply beyond the transition region. This behavior is observed in both benzene dimers and protein–ligand complexes, indicating that the fidelity of electrostatic fields is especially sensitive to under-resolution in COMPRESS. We find that the relationship between average grid loss and changes in interaction energies and molecular properties provides a practical guide for choosing *K*. In both protein–ligand complexes, |Δ*E*(*K*)| remains relatively small and changes gradually at low average grid loss, but begins to increase more rapidly in the 0.02–0.05 range. An average grid loss of 0.05 therefore, serves as a practical guideline for representation fidelity in the systems examined here. At the population level, 97% of GEOM-Drugs conformers remain below this value at *K* = 25, and the fraction decreases sharply with decreasing *K*, suggesting that *K* = 25 is sufficient for the large majority of conformers in this system.

Beyond field-preservation analysis, the COMPRESS decoder demonstrates the practical potential of the representation for molecular generation. Because the COMPRESS representation contains both spatial positions and physical attributes, the decoder can generate molecules that closely resemble or exactly match the target molecule. The validity of decoded structures depends on the amount and quality of information retained in the representation: both validity and the exact reconstruction rate increase with the resolution ratio *K*/*M*, rising from at least 23% at the lowest resolutions to about 93% at the highest. In addition, perturbing the representation produces a corresponding change in the decoded molecule. This shows that COMPRESS representations can be rationally modified to generate molecules with targeted structural changes.

Some limitations of the COMPRESS approach should be noted. First, unlike conventional coarse-grained models,^58,59^ COMPRESS is not designed to reduce a system’s molecular degrees of freedom to accelerate molecular dynamics simulations. COMPRESS does not provide a description of a molecule’s internal degrees of freedom or the associated energetic barriers for internal motions. Instead, it is designed to build molecular representations that reproduce spatially distributed molecular fields for a particular conformer. Because it does not explicitly encode chemical topology such as bonds, angles, and torsions, the representation alone does not guarantee atomistic realizability or chemical validity. However, as the representations allow for the natural calculation of nonbonded interactions, this approach can be used to generate rigid molecular models of arbitrary resolution for applications such as Brownian dynamics.^60^ Another limitation to consider is that the average grid loss should be treated as a practical indicator rather than a universal threshold. Because the grid loss is normalized relative to each molecule’s field magnitudes, identical numerical values may not reflect equivalent degrees of information loss across different systems. For example, in a molecule with a single strongly charged atom, that atom can dominate the electrostatic field magnitude and diminish the relative sensitivity of the normalized loss to errors elsewhere in the charge distribution. The decoder also has room for improvement. At the lowest resolutions, only 23% of decoded structures are valid, compared with the near-complete validity (>99%) reported for purely unconstrained 3D molecular generation.^32^ Unlike such models, which are trained at a single, fixed level of conditioning, our decoder is trained across all resolution ratios *K*/*M* within a single model; this wider range of task difficulty likely contributes to the lower validity at low *K*/*M*, where most of the valid structures are distinct from the reference rather than exact reconstructions. Validity also drops sharply when a COMPRESS representation is edited into an out-of-distribution (OOD) form not seen during training.

Addressing these limitations, we identify two directions for future work. First, the decoder’s performance could be improved using reinforcement learning, which recently has been used to steer molecular generation toward desired properties.^61,62^ A similar strategy could be applied here: the decoder could be rewarded for generating valid structures with low grid loss relative to the input COMPRESS representation. Second, we aim to generate molecules directly within a protein binding pocket. For example, this decoder could be combined with the Flexible Topology Pocket Explorer simulation framework,^30^ which produces a fixed number of ligand-like particles optimized within a protein pocket. In this setting, COMPRESS-like representations could be obtained in response to the binding pocket and then decoded into candidate molecular structures in a separate step. Such a strategy could make it possible to account not only for how a ligand fits within the pocket but also for how the surrounding protein environment may locally adapt with binding, which is not possible for conventional approaches that assume a rigid protein structure.

## Supplementary Material



## Data Availability

**Data and Software Availability:** Code to implement COMPRESS is available on GitHub at https://github.com/ADicksonLab/COMPRESS. The COMPRESS decoder is in a separate repository on GitHub at https://github.com/ADicksonLab/COMPRESS-Decoder.git. A Zenodo repository containing the compressed GEOM data set is available at 10.5281/zenodo.2007222. A second Zenodo repository containing the training data set for the COMPRESS decoder is available here: https://zenodo.org/records/21764574.

## Appendix

### A Detailed Description of $L_{\mathrm{restraint}}$

The restraint loss $L_{\mathrm{restraint}}$ is defined in eq 26 as a weighted combination of four loss terms:

26
$$L_{\mathrm{restraint}}\left(S,\;V\right)=\lambda_{\sigma }L_{\sigma }\left(V\right)+\lambda_{\mathrm{net}}L_{\mathrm{net}}\left(S,\;V\right)+\lambda_{\mathrm{overlap}}L_{\mathrm{overlap}}\left(V\right)+\lambda_{\mathrm{boundary}}L_{\mathrm{boundary}}\left(S,\;V\right)$$

Firstly, $L_{\sigma }\left(V\right)$ in eq 27 prevents *σ*_*k*_ from increasing above an upper bound:

27
$$L_{\sigma }\left(V\right)=\frac{1}{K}\overset{K}{\underset{k=1}{\sum }}{[\max\left(0,\;\sigma_{k}-\sigma_{\max}\right)]}^{2}$$

where *σ*_max_ = 4.0 Å was found to be appropriate. Second, $L_{\mathrm{net}}\left(S,\;V\right)$ in eq 28 preserves agreement of net-charge between the two sets of particles:

28
$$L_{\mathrm{net}}\left(S,\;V\right)={\left(\sum_{i=1}^{M}q_{i}^{S}-\sum_{k=1}^{K}q_{k}^{V}\right)}^{2}$$

where $q_{i}^{S}$ is the partial charge of atom *i* and $q_{k}^{V}$ is the charge parameter of site *k*. Thirdly, $L_{\mathrm{overlap}}\left(V\right)$ in eq 29 constrains sites from becoming too close to one another:

29
$$L_{\mathrm{overlap}}\left(V\right)=\frac{1}{N_{\mathrm{pair}}}\underset{i<j}{\sum }{[\max\left(0,\;w_{o}\sigma_{ij}-d_{ij}\right)]}^{2}$$

where $\sigma_{ij}=\frac{1}{2}\left(\sigma_{i}+\sigma_{j}\right)$ , *w*_*o*_ = 0.4, ∥*r*_*i*_ – *r*_*j*_∥_2_ is the distance between sites *i* and *j*, and *N*_pair_ = *K*(*K* – 1)/2.

Finally, an additional loss term $L_{\mathrm{boundary}}\left(S,\;V\right)$ in eq 30 is employed to prevent sites from drifting outside the boundary of *S*. This is used to prevent steric overlaps with atoms in the surrounding environment around *S*, such as protein or solvent atoms. The loss is defined as

30
$$L_{\mathrm{boundary}}\left(S,\;V\right)=\frac{1}{K}\overset{K}{\underset{k=1}{\sum }}{[\mathrm{ReLU}\left(w_{b}\sigma_{k}-d_{k}(S)\right)]}^{3}$$

where *w*_*b*_ = 0.5, $d_{k}\left(S\right)=\underset{b_{m}\in B\left(S\right)}{\min}{∥r_{k}-b_{m}∥}_{2}$ is the distance from site *k* to the closest boundary point in *B*(*S*) = {*b*_*m*_}. The boundary set *B*(*S*) is built by placing *n*_rays_ = 2048 points on a sphere surface large enough to cover all ${\{r_{i}\}}_{i=1}^{M}$ , and cast rays inward from the set *B* toward {*r*_*i*_} for each *i*. For each ray, we record the first intersection point with any atom-centered sphere of radius 0.5 *σ*_*i*_ as a boundary point *b*_*m*_. All boundary points are collected and then down-sampled to 5000 points using farthest-point sampling.^63^ Detailed workflow and an example set of boundary points are shown in Figure S1.

The weights λ_*σ*_ and λ_net_ are applied throughout optimization at λ_*σ*_ = 1.0 and λ_net_ = 0.1. In contrast, λ_overlap_ and λ_boundary_ are initialized at zero and increased gradually, allowing the sites to move freely during the initial optimization stage. Each weight is defined in eqs 31 and 32:

31
$$\lambda_{\mathrm{overlap}}\left(t\right)=\{\begin{array}{ll} 0, & t<15,\;\\ \min\left(0.005\left(t-15\right),\;0.1\right), & t\geq 15, \end{array}$$

32
$$\lambda_{\mathrm{boundary}}\left(t\right)=\{\begin{array}{ll} 0, & t<15,\\ 0.2, & 15\leq t<20,\\ \min\left(1.0,\;0.2\left(t-20\right)\right), & t\geq 20. \end{array}$$

In all runs, we use the optimization for 50 steps. These restraints are weighted such that they act as soft constraints rather than hard limits. Their contributions are small relative to $L_{\mathrm{grid}}$ , allowing sites to violate individual restraints when doing so substantially improves field agreement.

To ensure *σ* > 0 and *ϵ* > 0 during optimization, we do not optimize *σ* and *ϵ* directly. Instead, we optimize unconstrained variables *σ̃* and *ϵ̃* and map them at every step using *σ* = Softplus­(*σ̃*) + *δ* and *ϵ* = Softplus­(*ϵ̃*) + *δ*, with *δ* = 10^–3^. We therefore optimize the parameter set *θ* = {*r*, *q*, *σ̃*, *ϵ̃*}.

### B Details of Message Passing in COMPRESS Decoder

*G*_*V*_ represents the COMPRESS sites as a set of independent nodes, one per site, with no internal edges, such that each site is embedded independently without message exchange. Each site *k* is embedded from its invariant attributes into a scalar feature vector *u*_*k*_ = MLP_*V*_(*q*_*k*_, log *σ*_*k*_, log *ϵ*_*k*_), while its position *r*_*k*_ is preserved. This embedding is computed once at the beginning and remains constant. *G*_*V*_ influences *G*_*S*_ both by seeding the initial atom cloud *S*_0_ and by providing messages directly to each candidate atom in *G*_*S*_ during message passing.

*G*_*S*_ contains one node per candidate atom and fully connected edges between all atom pairs. Unlike *G*_*V*_, *G*_*S*_ is dynamic: it is initialized from *V*, iteratively updated through message-passing blocks, and ultimately read out to produce the reconstructed molecule. The initial atom cloud $S_{0}={\{(r_{i}^{\left(0\right)},\;q_{i}^{\left(0\right)})\}}_{i=1}^{M}$ is constructed as follows: each candidate atom *i* is assigned to a site *k*(*i*) ∈ {1, ..., *K*} by a balanced, randomly permuted round-robin assignment, and its initial position and charge are drawn according to eqs 33 and 34:

33
$$r_{i}^{\left(0\right)}=r_{k(i)}+0.5\sigma_{k(i)}\xi_{i},,\xi_{i}∼N\left(0,\;I_{3}\right)$$

34
$$q_{i}^{\left(0\right)}=\frac{q_{k(i)}}{n_{k(i)}}+\zeta_{i},,\zeta_{i}∼N\left(0,\;0.3^{2}\right)$$

where *n*_*k*(*i*)_ is the number of atoms assigned to site *k*(*i*), position noise *ξ*_*i*_ is resampled to enforce a minimum pairwise distance of 0.3 Å, and the constraint ∑_*i*: *k*(*i*)=*k*_ *ζ*_*i*_ = 0 for all *k* keeps the charges of the atoms assigned to each site summing to exactly *q*_*k*_.

Node and edge features are computed using eqs 35 and 36:

35
$$x_{i}={\mathrm{MLP}}_{S}\left(q_{i},\;\mathrm{emb}\left(a_{i}\right),\;\mathrm{emb}\left(t\right)\right)$$

36
$$e_{ij}={\mathrm{MLP}}_{E}\left(\mathrm{emb}\left(b_{ij}\right),\;\mathrm{RBF}\left(d_{ij}\right),\;d_{ij}/d_{\max},\;\mathrm{emb}\left(t\right)\right)$$

where emb­(*a*_*i*_) and emb­(*b*_*ij*_) are learned embeddings of the atom-type and bond-order tokens. emb­(*t*) is a standard sinusoidal embedding of the flow-matching time *t* ∈ [0, 1], shared by both features.^64^ RBF­(*d*_*ij*_) is a radial-basis expansion of the distance *d*_*ij*_ = ∥*r*_*i*_ – *r*_*j*_∥ between atoms *i* and *j*, defined in eq 37,

37
$$\mathrm{RBF}{\left(d\right)}_{m}=\exp\left(-{\left(\frac{d-c_{m}}{w}\right)}^{2}\right)$$

with centers *c*_*m*_ and width *w* as shown in eq 38 and B,

38
$$c_{m}=d_{\min}+\frac{m}{n_{\mathrm{rbf}}-1}\left(d_{\max}-d_{\min}\right)$$

39
$$w=\frac{d_{\max}-d_{\min}}{n_{\mathrm{rbf}}}$$

for *m* = 0, ..., *n*_rbf_ – 1, with *n*_rbf_ = 48, *d*_min_ = 0, and *d*_max_ = 12 Å.

*G*_*S*_ is then iteratively updated through six message-passing blocks: three large blocks followed by three small blocks. The large blocks are made up of two layer-level steps: a *V* → *S* layer, in which atoms receive messages from the *K* COMPRESS sites, and an *S* → *S* layer, in which atoms exchange messages among themselves. A small block runs the *S* → *S* layer only, letting the network refine geometry and connectivity in the later blocks. Each function denoted *ϕ* below is a two-layer neural network that maps its inputs to a new feature vector.

Within the *V* → *S* layer of a large block, each atom first computes a gated message from every site using eqs 40–42,

40
$${\mathrm{m̃}}_{i\leftarrow k}=\phi_{V\to S}\left(x_{i},\;u_{k},\;\mathrm{RBF}\left(d_{ik}\right),\;d_{ik}/d_{\max}\right)$$

41
$$g_{ik}=\sigma \left(\phi_{\mathrm{gate}}\left({\mathrm{m̃}}_{i\leftarrow k}\right)\right)$$

42
$$m_{i\leftarrow k}=g_{ik}{\mathrm{m̃}}_{i\leftarrow k}$$

where *σ* is the sigmoid function, squashing the gate *g*_*ik*_ into (0, 1) so that each site’s message can be passed through or suppressed. These gated messages are aggregated into a single summary, $c_{i}=\frac{1}{K}\overset{K}{\underset{k=1}{\sum }}m_{i\leftarrow k}$ . Node features and atom positions are then updated in place using eqs 43 and 44:

43
$$x_{i}\leftarrow \mathrm{LN}\left(x_{i}+\phi_{\mathrm{node}}\left(x_{i},\;c_{i}\right)\right)$$

44
$$r_{i}\leftarrow r_{i}+\frac{\alpha }{K}\overset{K}{\underset{k=1}{\sum }}\frac{r_{i}-r_{k}}{d_{ik}+1}\phi_{w}\left(m_{i\leftarrow k}\right)$$

where LN denotes layer normalization and *α* = 0.1. Finally, an edge-feature update revises every atom–atom edge using the newly updated positions and node feature as shown in eq 45,

45
$$e_{ij}\leftarrow \mathrm{LN}\left(e_{ij}+\phi_{E}\left(e_{ij},\;x_{i},\;x_{j},\;\mathrm{RBF}\left(d_{ij}\right),\;d_{ij}/d_{\max}\right)\right)$$

The *S* → *S* layer applies the same message/gate/update process among candidate atoms themselves, with its own and separately learned functions. The one difference is that its message additionally conditions on the edge feature *e*_*ij*_, since *G*_*S*_ maintains edges between atoms.

## Notes

A depiction of boundary dots used for the boundary loss computation; multiple stable states obtained for benzene at different *K* values; a zoomed-in view of the normalized grid loss for the 20 scaffold molecules; average grid loss versus changes in global molecular properties; normalized grid loss components for ligand-protein complexes (PDF)
