# Supplementary material for: Building a Physically Interpretable Compressed Chemical Space via Grid-Based Optimization
Source: J Chem Inf Model. 2026 Oct 15;66(20):13413–31. doi: 10.1021/acs.jcim.6c01542 (PMC13715499; doi:10.1021/acs.jcim.6c01542)
Supplement: Supplementary file 1 [file ci6c01542_si_001.pdf]

Supplementary Information: Building a Physically-Interpretable Compressed Chemical Space via Grid-Based Optimization

Seungmin Yoon[†] and Alex Dickson[*,‡,¶]

†*Department of Pharmacology and Toxicology, Michigan State University, East Lansing, Michigan 48824, USA*

‡*Department of Computational Mathematics, Science and Engineering, Michigan State University, East Lansing, Michigan 48824, USA*

¶*Department of Biochemistry and Molecular Biology, Michigan State University, East Lansing, Michigan 48824, USA*

E-mail: alexrd@msu.edu

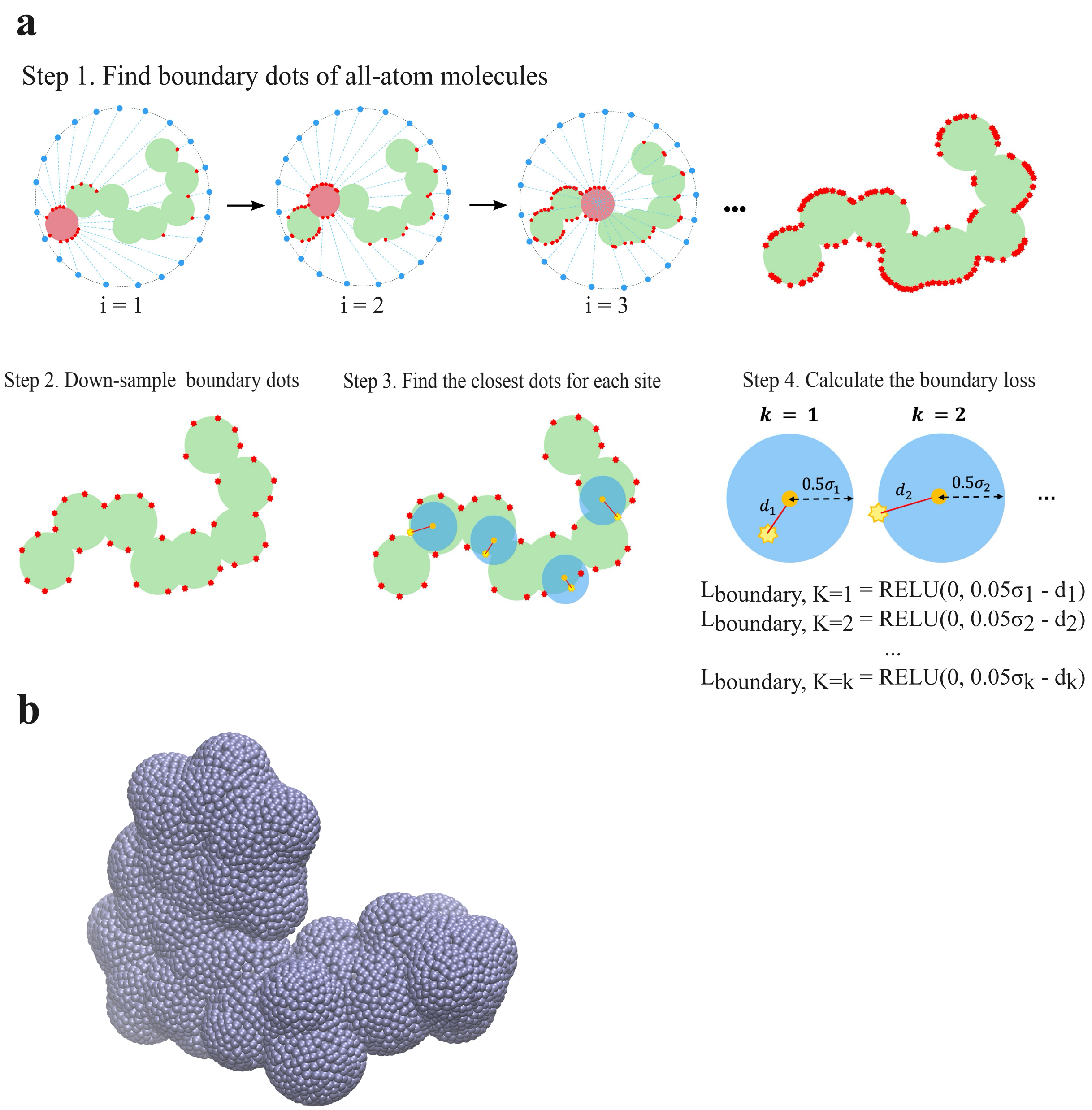

Figure S1: Boundary-loss calculation and an example of boundary-dot definition. (a) Schematic illustration of how boundary loss is computed from boundary dots of the all-atom molecule and their distances to COMPRESS sites. (b) Boundary-dot definition obtained for the carazolol in the B2AR complex structure (PDB:2RH1).

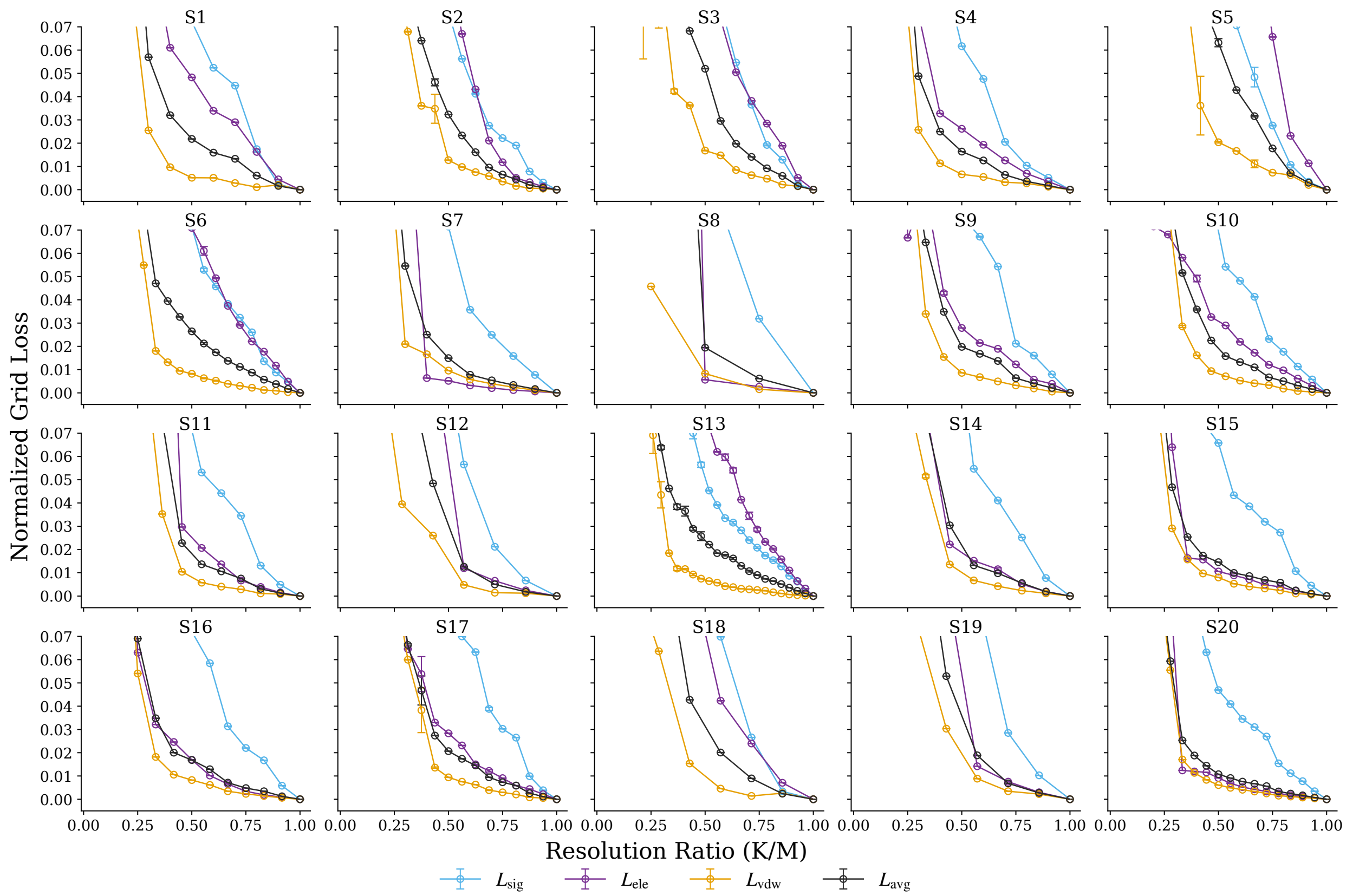

Figure S2: Zoomed-in view of the normalized grid loss across 20 scaffold molecules as a function of resolution ratio, K/M, with the y-axis restricted to $L_{\text{avg}} < 0.07$ to highlight the low-loss region. Each subplot corresponds to one of the scaffold molecules S1-S20 labels shown in Fig. 4.Each figure corresponds to a single molecule in Fig. 4. The average grid loss, L_{avg}, was calculated as the mean of six components: L_{sig}, L_{ele}, L_{pos}, L_{neg}, L_{rep}, and L_{att}. The vdW grid loss, L_{vdW}, is defined as $(L_{\text{rep}} + L_{\text{att}})/2$.

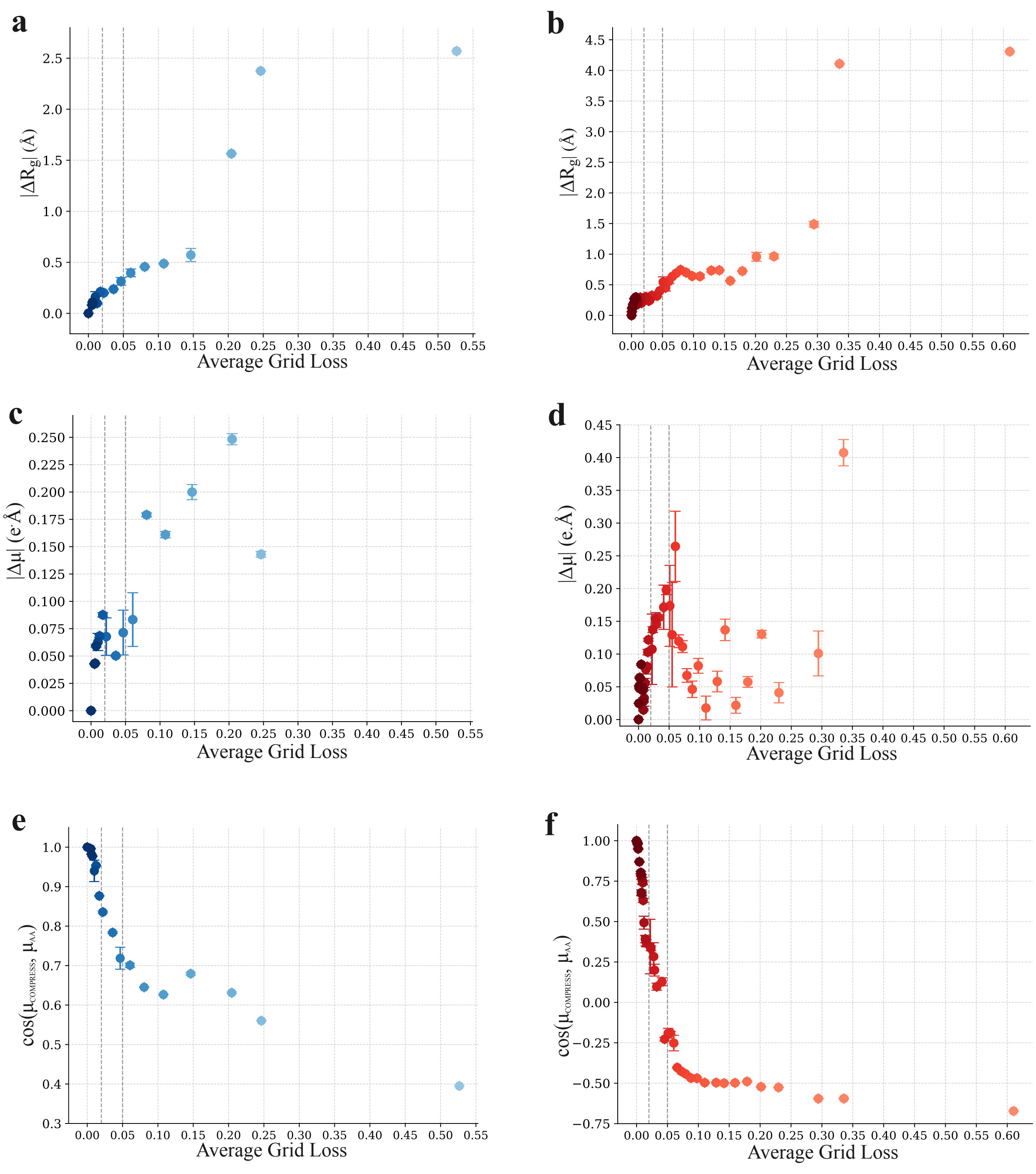

Figure S3: Average grid loss versus changes in global molecular properties across different K. The left column (a, c, e) shows results for trypsin and the right column (b, d, f) for B2AR. Top row: absolute error in radius of gyration ($|\Delta R_g|$). Middle row: absolute error in dipole moment ($|\Delta\mu|$). Bottom row: dipole direction cosine similarity. Darker colors indicate larger K.

a

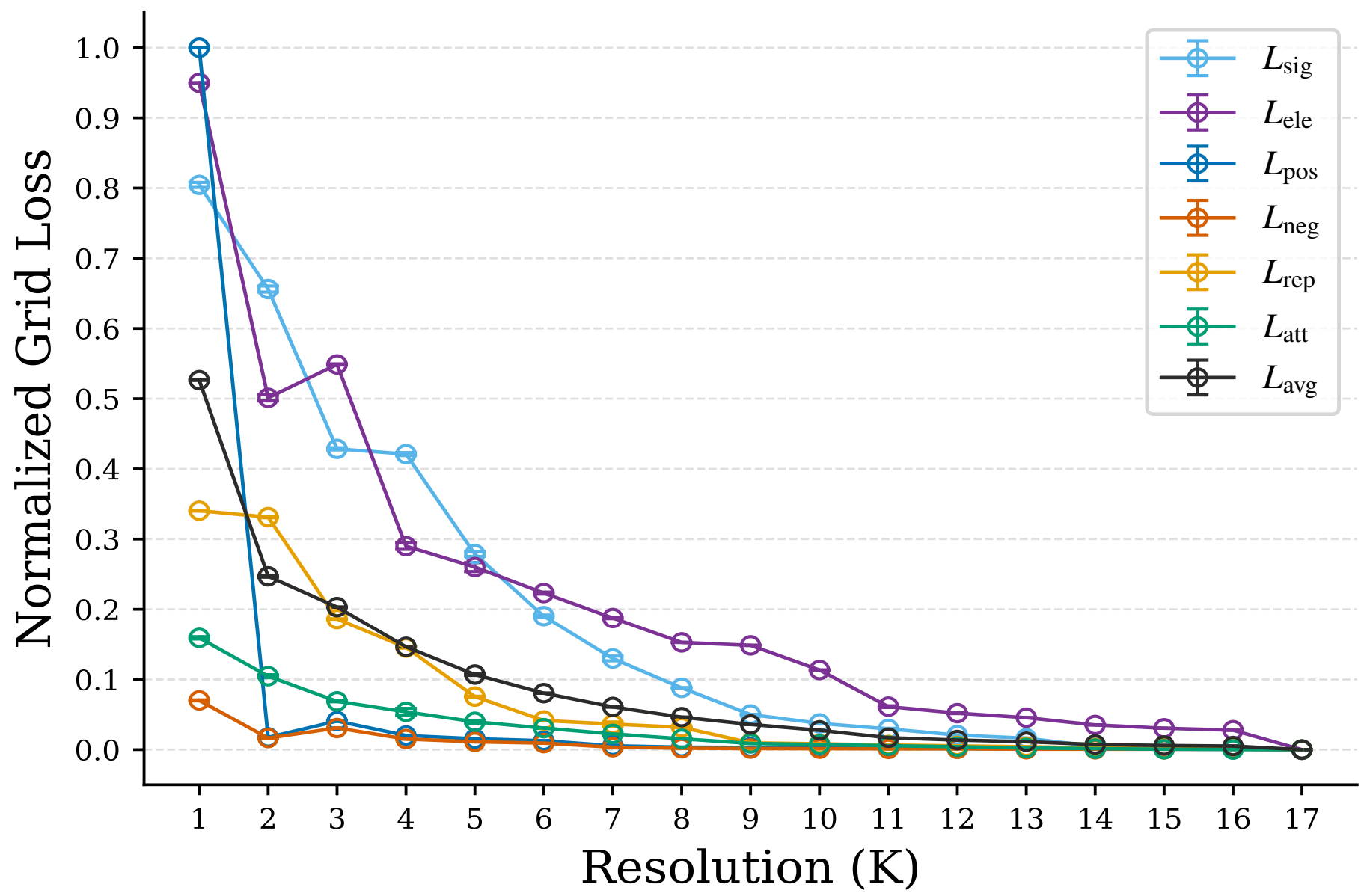

b

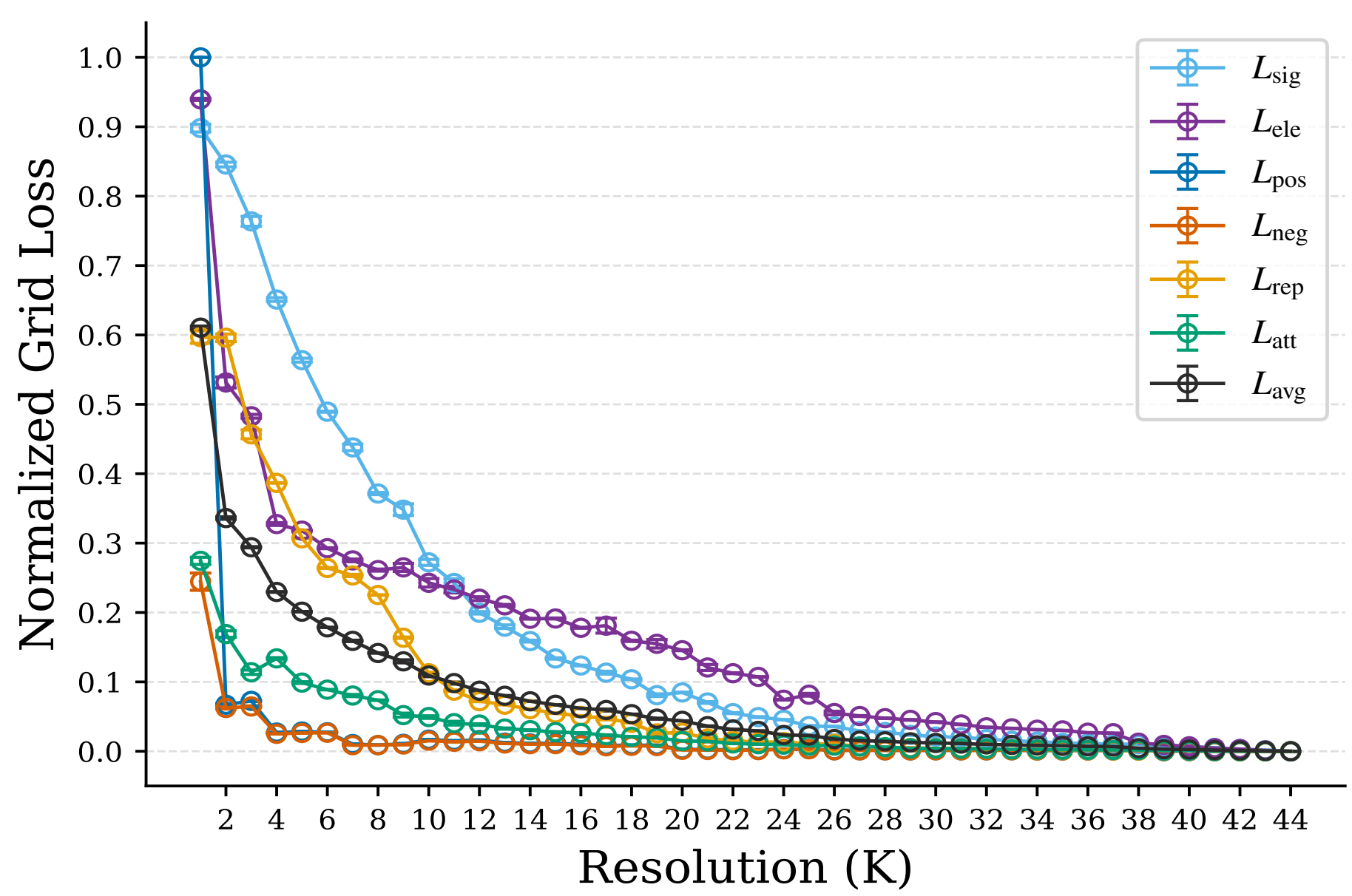

Figure S4: Normalized grid loss for COMPRESS representations of the ligands in (a) trypsin and (b) B2AR as a function of K. The average loss, L_{avg}, represents the mean of the six loss components (L_{sig}, L_{ele}, L_{pos}, L_{neg}, L_{rep}, and L_{att}).

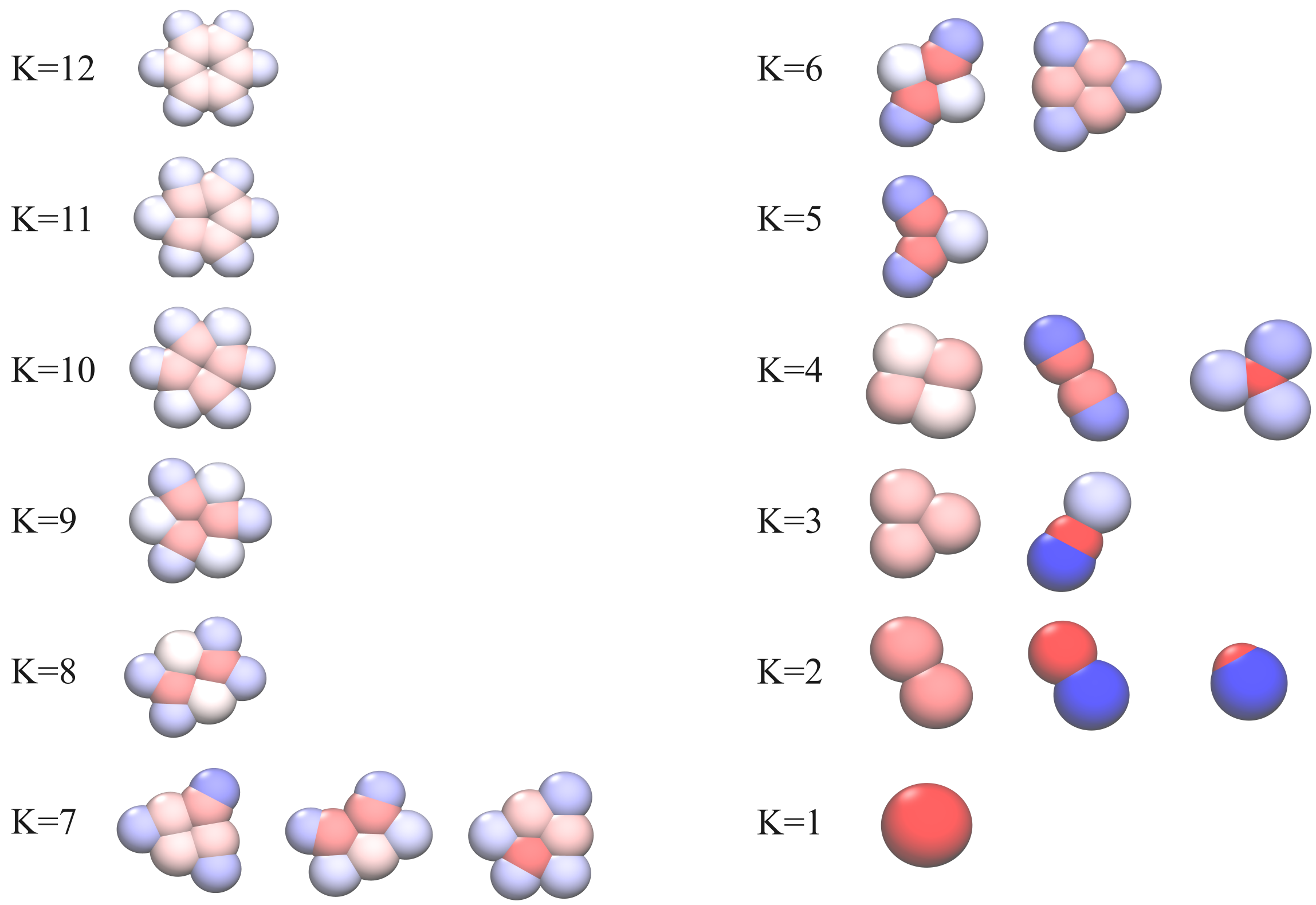

Figure S5: COMPRESS representations of benzene at different resolutions K, obtained from 10 independent optimization runs. The top three distinct representations are shown, ordered by the frequency observed. Sites are colored by charge (red: negative, blue: positive) and scaled by LJ diameter σ.
